# Profiling of secondary metabolite and evaluation of anti-diabetic potency of *Crotalaria quinquefolia* (L): *In-vitro*, *in-vivo*, and *in-silico* approaches

**DOI:** 10.1016/j.jsps.2023.101887

**Published:** 2023-11-25

**Authors:** Nazmun Nahar, Md. Nazmul Hasan Zilani, Partha Biswas, Md. Morsaline Billah, Shabana Bibi, Norah A. Albekairi, Abdulrahman Alshammari, Md. Nazmul Hasan

**Affiliations:** a Laboratory of Pharmaceutical Biotechnology and Bioinformatics, Department of Genetic Engineering and Biotechnology, Jashore University of Science and Technology, Jashore 7408, Bangladesh; b Department of Pharmacy, Jashore University of Science and Technology, Jashore 7408, Bangladesh; c Biotechnology and Genetic Engineering Discipline, Khulna University, Khulna 9208, Bangladesh; d Department of Biosciences, Shifa Tameer-e-Millat University, Islamabad 41000, Pakistan; e Yunnan Herbal Laboratory, College of Ecology and Environmental Sciences, Yunnan University, Kunming 650091, China; f Department of Pharmacology and Toxicology, College of Pharmacy, King Saud University, Post Box 2455, Riyadh 11451, Saudi Arabia

**Keywords:** *Crotalaria quinquefolia*, Antidiabetic activity, QSAR study, Enzyme activity, Computational study

## Abstract

Traditional medicinal plants have played a promising role in the human health system. In folklore medicine, *Crotalaria quinquefolia* L. is used to treat fever, pain, eczema, impetigo, lung infections, scabies. The present investigation was executed to identify secondary metabolites responsible for anti-diabetic potential of *C. quinquefolia* L. leaf extract along with their possible mechanistic pathways. The anti-hyperglycemic activity was assessed by *in vitro* α-amylase and α-glucosidase inhibitory assays and an *in vivo* oral glucose tolerance test and diabetogenic effect of streptozotocin in mice, followed by an integrative computational analysis. A total of 23 compounds were identified through GCMS and HPLC. The extract showed potent *in-vitro* α-amylase and α-glucosidase suppressive activity with IC_50_ values of 12.8 ± 0.1 µg/mL and 36.3 ± 0.07 µg/mL, respectively. In an *in vivo* oral glucose tolerance test, the extract (400 mg/kg body weight) prompted blood glucose levels to plummet by 18.9 % after 30 min, compared to the normal control and streptozotocin induced diabetes test, maximum glucose reduction was observed 11.67 % by dose of 200 mg/kg compared to the control; glibenclamide and extract (400 mg/kg) reduced blood glucose levels by 1.3 % and 16.7 %, respectively, compared to diabetic control at the end of the trial. Additionally, among the identified compounds, myricetin, quercetin, rutin, and kaempferol revealed good binding affinity as well as stability with the studied anti-diabetic proteins in docking and molecular dynamics simulation studies. Furthermore, QSAR analysis and network pharmacology studies of the identified compounds divulged enhanced insulin secretion stimulation, insulin receptor kinase activity, PPARγ expression; enzyme inhibition (α-glucosidase, α-amylase) and protection of the pancreas -mediated antidiabetic effects. Besides, they proved strong inhibitory potential against the studied antidiabetic proteins in other computational analysis. Based on the present findings, it can be affirmed that *C. quinquefolia* extract possesses anti-diabetic activity.

## Highlights

• Identification of bioactive compounds through HPLC, GCMS.
• *In vitro* antioxidant activity assay with content determination of secondary metabolites.
• *In vitro* α-amylase and α-glucosidase inhibitory assays.
• *In vivo* anti-diabetic activity in mice model.
• *In vitro* and *In vivo* toxicity assay.
• *In silico* molecular docking, molecular dynamic simulation, MMGBSA and Network Pharmacology analysis.

## Graphical abstract

**Figure:**
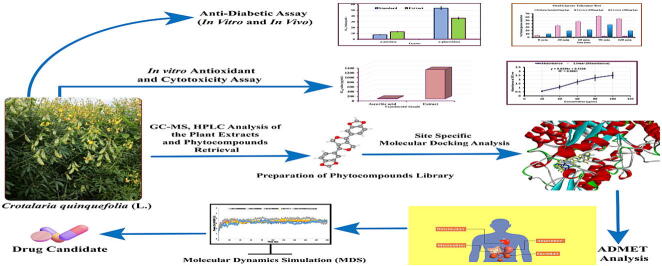

## 1 Introduction

Diabetes mellitus, a particularly common endocrine illness, causes blood sugar levels to rise either because beta cells aren't producing enough insulin (Type 1) or the insulin that is generated is rendered useless (Type 2). It is brought on by a problem with insulin resistance, production, or both, which affects how proteins, fats, and carbohydrates are metabolized (Ahmed et al., 2019). A prolonged state of hyperglycemia triggered by diabetes has previously been linked to, over an extended period, the destruction of organs, malfunction, and ultimately organ dysfunction, particularly in the kidneys, eyes, nerves, heart, and blood vessels. Even though there has been a lot of research done to control diabetes and a number of oral medications have been established, the field of science is still looking into developing novel oral diabetic treatments because of current agents' limitations (Bhavsar et al., 2009). Antioxidants have been speculated to play a key role in treating diseases like diabetes mellitus. It has been reported that the generation of free radicals in the cells as a result of oxidative stress could lead to diabetes, especially type 2.

The onset of diabetes is closely associated with oxidative stress, mainly through oxidation, nonenzymatic protein glycation, and oxidative degradation of glycated proteins. Reactive oxygen species (ROS) are continuously formed during energy production by cells using the essential cellular metabolism ingredient oxygen. Reactive oxygen species (ROS) are capable of oxidizing cellular proteins, nucleic acids, and lipids, which increases in both types of diabetes. Elevation of ROS such as mitochondrial superoxide in endothelial cells and endoplasmic reticulum stress, followed by reduced antioxidant defence mechanisms, provokes cellular and enzyme damage and lipid peroxidation, which subsequently lead to the development and progression of insulin resistance by disrupting insulin receptor signal transduction and insulin deficiency by attacking insulin-producing cells (Khouya et al., 2022, Sarian et al., 2017).

At high concentrations, ROS causes oxidative stress and spoils important cellular structures and activities, including diabetes (Maruhashi & Higashi, 2021). Overproduction of ROS and a diminished antioxidant status resist insulin sensitivity, beta-cell malfunction, and increased glucose intolerance, which ultimately trigger diabetes (Black, 2022). Both interior and exterior antioxidants act primarily as free radical scavengers, mitigating and mending harm created by ROS (Ahmadi et al., 2023). Antioxidants, including vitamins, dietary supplements, and various elements of plants, have been designed to mitigate the effects of oxidative stress in diabetes. Modern synthetic antidiabetic medications, in addition to lifestyle modifications are prime ways to regulate diabetes mellitus (Mechchate et al., 2021). However, using these medications was linked to a variety of adverse effects. As a result, maintaining diabetes without encountering any adverse effects is indeed difficult. The medications based on plant phytochemicals have better effects with fewer negative effects (Quranayati et al., 2022).

Hyperglycemia has been treated with a variety of plant species. Hence, plants could be a potential strategy for alleviating or deterring high blood sugar levels and other catastrophic illnesses brought on by oxidation products. Therefore, finding novel bioactive molecules with stronger anti-diabetic action and fewer side effects is urgently needed. It is still ongoing to look for drugs from natural resources with innovative qualities to treat diabetes mellitus (Tan et al., 2019). Bangladesh is a natural hub for medicinal plants. About 500 medicinal plants are traditionally used by many Bangladeshi people for their healthcare requirements (Ghani, 2005). In traditional medical practices, *Ageratum conyzoides*, *Andrographis paniculate*, *Azadirachta indica*, *Clitoria ternatea*, *Musa sapientum,* Swertia *chirata*, *Terminalia arjuna*, and *Trigonella foenum-graecum* are used in diabetes mellitus (Ahmed et al., 2023).

*Crotalaria quinquefolia L*., of the Fabaceae family, is a perennial plant. Traditionally, it is used to treat fever, pain, eczema, impetigo, lung infections, scabies (Jahan et al., 2019). It also possesses antibacterial properties (Ghani, 2005, Jahan et al., 2019). The seed of *C. quinquefolia* possesses high levels of pyrrolizidine alkaloids. The plants of the Fabaceae family revealed antimicrobial, anti-nociceptive, anti-diabetic, anti-inflammatory, anticancer, and antioxidant activities, and they also possess biologically active substances such as tannins, flavonoids, alkaloids, terpenes (Devendra et al., 2012, Jahan et al., 2019). Our understanding is that *C. quinquefolia* has been the subject of a relatively small number of investigations. Therefore, the main objective of the current work was to seek out secondary metabolites using HPLC and GCMS studies as well as their antidiabetic mechanisms using *in vitro*, *in vivo*, and *in silico* analyses.

## 2 Methods and materials

### 2.1 Chemicals and reagents

Gallic acid, 3,4-Dihydroxybenzoic acid, Catechin hydrate, Catechol, (-) Epicatechin, Caffeic acid, Vanillic acid, Syringic acid, Rutin hydrate, *p*-Coumaric acid, *trans*-Ferulic acid, Rosmarinic acid, Myricetin, Quercetin, *trans*-Cinnamic acid, and Kaempferol were purchased from Sigma-Aldrich (St. Louis, MO, USA). Acetonitrile (HPLC), methanol (HPLC), acetic acid (HPLC), sodium carbonate, aluminum chloride, potassium acetate, ursolic acid, perchloric acid, vanillin, glacial acetic acid, DPPH, ascorbic acid, ferric chloride, TPTZ, potassium ferricyanide, trichloroacetic acid, acarbose, starch, HCl, alpha amylase, alpha glucosidase, glibenclamide, vincristine sulphate, and ethanol were obtained from Merck (Darmstadt, USA). All other chemicals were reagent-grade.

### 2.2 Experimental animals

We obtained albino mice of both sexes from the International Centre for Diarrheal Disease Research in Bangladesh (ICDDR, B) that weighed 20–30 g and were 6–8 weeks old. These mice were given 10 days to become acclimated to the laboratory environment, which consisted of a clean, perishable cage at 24 °C, 50–70 % relative humidity, and a 12-hour light/12-hour dark cycle and supplied with a standard feed and water precisely. The Ethical Review Committee, Faculty of Biological Science and Technology, Jashore University of Science and Technology [Ref: ERC/FBS/JUST/2022–111], provided the ethical standard for all animal model experiments, which were conducted in conformity with the European Community norms (EEC directives of 1986; 86/609/EEC).

### 2.3 Preparation of crude extract

The leaves of *C. quinquefolia* L. were collected at November 28, 2020 from Jashore, Bangladesh (23°13′57.8″N 89°07′35.1″E), and verified from the Bangladesh National Herbarium (voucher specimen: DACB 63700). The shade-dried powder was immersed in 98 % ethanol for fifteen days in an air-tight container and stirred frequently. Then, the filtrate was dried at rotary evaporator (RE-100 PRO, DLAB Scientific Inc., China) and the yield of the extract was 2.86 % (w/w). The extract was kept at 4 °C until the experiment began.

### 2.4 Total polyphenol content

The total phenol content was estimated using the Folin-Ciocalteu technique (Muflihah et al., 2021). The extract and standard were treated with sodium carbonate (7.5 % w/v) and the Folin-Ciocalteu reagent (1:10 v/v). At 750 nm, the absorbance was checked following an incubation of 30 min at 40 °C (Shimadzu UV visible spectrophotometer, Japan). The total phenol content of the extract was calculated in mg gallic acid equivalent per gram of dry extract.

### 2.5 Total flavonoid content

The total flavonoid content (TFC) was substantiated as mg quercetin equivalent (QE)/g of dry extract using a standard quercetin calibration curve (Zilani et al., 2021). A mixture of aluminium chloride (10 % w/v), potassium acetate (1 M), and distilled water was incubated for 5 min to deliberate absorbance at 510 nm.

### 2.6 Total terpenoid content

Ursolic acid was used to construct the standard calibration curve (B. Biswas et al., 2021). Initial mixtures of extract, perchloric acid (1 mL), and vanillin/glacial acetic acid (5 % w/v) were added to glacial acetic acid to assess the absorbance at 548 nm. The results were portrayed as mg ursolic acid/g extract.

### 2.7 Identification of the polyphenol compounds by HPLC

With slight tweaks from the techniques previously disclosed (Ahmed et al., 2021). HPLC-DAD analysis was used to detect and quantify a subset of the plant extracts' polyphenolic components. HPLC investigation was conducted using a Shimadzu (LC–20 A, Japan) implementing a column oven (CTO–20 A), auto sampler (SIL–20A HT), photodiode array detector (SPD–M20 A), and binary solvent delivery pump. A Luna C_18_ (5 µm) Phenomenex column (4.6 x 250 mm) was used for separation, which was carried out at 33 °C. In this investigation, the following gradient elution periods were used: 0.01–20 min (5–25 % A), 20–30 min (25–40 % A), 30–35 min (40–60 % A), 35–40 min (60–30 % A), 40–45 min (30–5 % A), and 45–50 min (5 % A). The mobile phase consisted of A (1 % acetic acid in acetonitrile) and B (1 % acetic acid in water). The flow rate was fixed at 0.5 mL/min and 20 L of sample was infused. The UV detector was executed with a setting of 270 nm in order to validate the approach and assessment. The mobile phase was degassed under vacuum after being filtered using a 0.45 m Nylon 6, 6 membrane filter (India). For the creation of the standard calibration, a typical stock solution containing the following ingredients was made in methanol: gallic acid (20 g/mL); 3,4-Dihydroxybenzoic acid (15 g/mL); catechin hydrate (50 g/mL); catechol, (-) epicatechin, and rosmarinic acid (30 g/mL each); caffeic acid, vanillic acid, syringic acid, rutin hydrate, p-Coumaric acid, *trans*-ferulic acid, quercetin (10 µg/mL each); myricetin, kaempferol (8 µg/mLeach); *trans*-cinnamic acid (4 µg/mL).

### 2.8 Gas chromatography mass spectroscopy (GCMS)

GCMS evaluation was accomplished utilizing a column (Elite-35, 30 m length, 0.25 mm diameter, 0.25 m thickness of film), a Claruso R690 gas chromatograph (PerkinElmer, CA, USA), and a Clarus SQ 8C mass spectrophotometer (PerkinElmer, CA, USA). One microliter sample was infused with pure helium (99.999 %) as the carrier gas in splitless at a flow rate of 1 mL/min for 40 min. To characterize the material, high-intensity electron ionization (70 eV) was deployed. The column oven was warmed to 60 °C for 1 min, escalated by 5 °C /min to 240 °C, and retained at that temperature for 4 mins while the intake temperature was held constant at 280 °C (Munshi et al., 2022). The sample components might be recognized by comparing them to the National Institute of Standards and Technology (NIST) database.

### 2.9 Antioxidant activity

#### 2.9.1 DPPH radical scavenging assay

To ascertain the extract's capacity to scavenge DPPH radicals, a stock solution of 1024 g/mL was created. Each concentrated sample solution (512–1 µg/mL) was introduced into the 0.004 % DPPH solution. After incubating in the dark, the absorbance was recorded at 517 nm. The following formula was used to calculate the DPPH scavenging potential: Scavenging (%) = [1- (A_Sample/standard_/A_control_] × 100. This information was used to compute the IC_50_ value and contrast it to ascorbic acid (Bristy et al., 2022).

#### 2.9.2 Ferric reducing antioxidant power (FRAP)

A modified version of the FRAP test was used to measure the ferric reducing antioxidant power value as mg ascorbic acid/g of dry extract (Naji et al., 2020). The FRAP reagent is composed of 10 mM TPTZ in 40 mM HCl, 20 mM ferric chloride, and 300 mM acetate buffer (pH 3.6) at 1:1:10 (v/v/v) ratios. Diluted ascorbic acid was combined with the FRAP reagent. At 593 nm, absorbance was measured following incubation at 37 °C.

#### 2.9.3 Reducing power assay

To calculate the reducing power value, mixtures of the extracts, standard, phosphate buffer (0.2 M, pH 6.6), and potassium ferricyanide (1 % w/v) were stirred at 50 °C for 20 min. After adding trichloroacetic acid (10 % w/v), the solution was centrifuged at 3000 rpm for 10 min. Ferric chloride (0.1 % w/v) and distilled water were added to the supernatant. At 700 nm, absorbance was assessed after 10 min (Can-Cauich et al., 2017).

### 2.10 *In vitro* anti-diabetic activity assay

#### 2.10.1 Alpha-amylase inhibitory activity

Alpha-amylase inhibitory activity of plant material was conducted in a microtiter plate with a little modification (Salahuddin et al., 2020). Mixtures of sodium phosphate buffer (0.02 M, pH 6.9), enzyme solution (1 unit/mL), and extract were incubated at 37 °C. As a positive control, acarbose was utilized. Thereafter, add starch (0.25 % w/v) and incubate at 37 °C. Mixing HCl (1 M) solution followed by the addition of iodine reagent. A microplate reader was used to measure the absorbance at 620 nm. The IC_50_ value represented the inhibitory action against alpha amylase.

#### 2.10.2 Alpha-glucosidase inhibitory activity

The alpha-glucosidase inhibitory potency was characterized by measuring the formation of 4-nitrophenol with some modifications (Boulfia et al., 2021). Thus, the mixture of extracts and alpha glucosidase (1 unit/1mL) was preincubated at 37 °C. Subsequently, a pNPG solution (0.1 mM) in sodium phosphate buffer (0.1 M, pH 6.7) was added and incubated. After adding sodium carbonate (0.1 M), the absorbance was measured at 405 nm. Acarbose served as a positive control in this study. From these data IC_50_ value was calculated.

### 2.11 *In vivo* anti-diabetic activity assay

#### 2.11.1 Oral glucose tolerance test

The fasted mice were separated into four sets: control (distilled water), standard (glibenclamide, 5 mg/kg body weight), and extract (200 and 400 mg/kg body weight), respectively. Then, single dose of distilled water, standard drug and extract were orally administered to the specific treatment group. After that, blood specimens were obtained from the tail vein at experiment period of 0, 30, 60, 90, and 120 min after the consumption of oral glucose (3 g/kg). The blood glucose levels were examined using glucose test strips and a glucose meter (ACCU-CHECK Active, Roche Diabetes Care South Africa (Pty) Ltd., South Africa) (Claro-Cala et al., 2020).

#### 2.11.2 Diabetogenic effect of streptozotocin in mice

A single intraperitoneal injection of 60 mg/kg body weight of streptozotocin (STZ), freshly dispersed within cold citrate buffer (0.1 M, pH 4.5), was administered to the overnight starved mice. The mice were split into five groups: and control (not treated), standard (glibenclamide 5 mg/kg), diabetic control (streptozotocin treatment) and extract (200 and 400 mg/kg body weight) respectively. The specific treatment has been orally administered to the specific group of mice for 21 days. Daily, the newly prepared solutions were consumed orally. On days 1, 7, 11, 15, and 21, overnight-fasted animals had their blood sugar levels checked using a glucometer. On the 21st day the effects of the extract on diabetic mice were assessed (Gupta et al., 2012).

### 2.12 Toxicity test

The *in vivo* toxicity test was performed using the brine shrimp *Artemia salina* (Niksic et al., 2021). The pre-hatched nauplii were placed in each calibrated test tube containing varying diluted solutions of the extracts. The concentration that was minimal enough to cause 50 % mortality (LC_50_) was determined by counting the number of dead larvae. Moreover, an acute oral toxicity test was carried out on mice at doses of 1000, 2000, and 4,000 mg/kg body weight of extract in order to evaluate the acute toxicity. The treatment groups were observed for 14 days (El Ouahdani et al., 2021).

### 2.13 *In silico* analysis

#### 2.13.1 Molecular docking and post docking visualization

A library of 23 phytochemicals was detected by GC–MS and HPLC analysis of the extracts. The Pub Chem database was used to get the name, Pub Chem ID, molecular weight, and structure of those compounds. ADMET profiling was carried out to assess the pharmacokinetic features of phytochemicals using the web programs pk^CSM^ and Swiss ADME (Biswas et al., 2022, Biswas et al., 2021, Dey et al., 2022). 1B2Y, 3WY1, 6JB3, and 8B8Z have been specified as the targeted receptors and the existing blockers of alpha-amylase, alpha glucosidase, sulphonyl urease, and the peroxisome proliferator-activated gamma receptor, respectively. Several bioactive phytochemicals were taken into consideration for this investigation, and as standard references, acarbose, miglitol, and repaglinide were used. The protein was constructed using Maestro Desmond's Protein Preparation Wizard, version 12.5 (Schrödinger Release 2020–3 Schrödinger, LLC, New York, NY, 2020). After that, the protein's natural ligand was targeted in order to create the receptor grid. Using Maestro (Schrödinger Release 2021–2: Maestro, Schrödinger, LLC, New York, NY, 2020–3), the molecular docking was carried out (Andalib et al., 2023, Hasan et al., 2022, Jabin et al., 2023).

#### 2.13.2 Molecular dynamic simulation

The molecular dynamic simulation was performed using 100 ns of the protein–ligand complex structures in the Linux environment using the “Desmond v3.6 Program” in Schrödinger (Dey et al., 2022, Hasan et al., 2022, Hasibuzzaman et al., 2023, Khan et al., 2023). After building solvency protein systems with ligand complexes, the system framework was reduced and relaxed utilizing the protocol carried out using force field constants OPLS3e included inside the Desmond package. The Simulation Interaction Diagram (SID) of the Desmond modules provided in the Schrödinger suite was used to assess the fidelity of the MD simulation during the whole simulation event. The stability of the protein–ligand complex system was evaluated using the RMSD, RMSF, protein–ligand contacts (P-L), intramolecular hydrogen bonds, SASA, Rg, MolSA, and PSA measurements.

#### 2.13.3 Post simulation binding energy calculation (MM/GBSA)

The relative binding free energies (ΔG_bind) were computed using the thermal_mmgbsa.py Python script during the 100 ns simulation deploying the Molecular Mechanics Generalized Born Surface Area (MM/GBSA) module in Schrodinger. The Desmond MD trajectory was divided into 20 independent frame snapshots, each of which was run through MMGBSA before the ligand and receptor were separated. Binding energy is calculated as follows: ΔG_bind_ = ΔE_MM_ + ΔG_solv_ + ΔG_SA_ (ΔG_solv_ referred to the energy difference between the complex's GBSA solvation energy and the total of the ligand and protein solvation energies, ΔG_SA_ referred to the difference in energy between the complex's surface area and the total of the protein and ligand's surface area energy, where ΔE_MM_ is the difference in minimized energies as following: ΔE_MM_ = E_(complex) –_ E_(ligand)– _E_(receptor)_). The MM/GBSA was run on Maestro with default options, which use the 1.0 dielectric constant by using the thermal_mmgbsa.py script on the MD trajectory (Akash et al., 2023, Zamzami, 2023).

#### 2.13.4 Ligand activity prediction by QSAR analysis

The biological activity of the 23 phytochemicals was contrasted using the renowned server PASS (Prediction of Activity Spectra for Substances), which forecasts results depending on a substance's structural makeup. The possibility that a certain substance will be a member of both the active and inactive subgroups of that substance is predicted using the Structure Activity Relationship Base (SAR Base). The input was provided in SMILES (Simplified Molecular Input Line Entry System) format for the phytochemical structure. The Pa (probable activity) and Pi (probable inactivity) values were determined for each ligand. Consequently, only activities involving diabetes were taken into account (Filimonov et al., 2014).

#### 2.13.5 Network pharmacology

To identify the co-expressed and overexpressed genes that regulate the expression of the targeted proteins in diabetic patients, the four chosen protein-mediated string networks were created. First, the STRING database (https://string-db.org) was used to extract all of the receptor protein strings. Cytoscape 3.8.2 (https://cytoscape.org/), which is run through the Java Runtime Environment (https://www.oracle.com/java/technologies/downloads/), was used to identify and describe the distinct nodes and edges of the protein. Second, in order to fully comprehend the potential drug metabolism, an interacting network between the drug candidates and their four protein targets was constructed using STITCH and Cytoscape 3.8.2. The lists of several diabetes-related proteins were generated using the canonical SMILES of medication candidates entered into the STITCH. The protein list was then entered into the Cytoscape 3.8.2 program to create an original drug-protein interaction (DPI) network that included the drug candidates and several additional diabetic protein targets that the medications can affect (Morshed et al., 2022).

### 2.14 Statistical analysis

The data are displayed as mean ± standard deviation (S.D.) after each experiment was independently performed at least three times. When p < 0.05, differences were deemed statistically significant.

## 3 Research findings

### 3.1 Chemical profiling

**Fig. 1:**
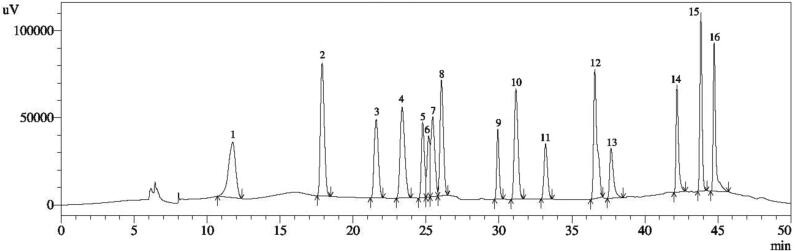
HPLC fingerprint standard mixture of polyphenolic compounds (Peak 1: Gallic acid; Peak 2: 3,4 dihyrdoxybenzoic acid; Peak 3: Catechin hydrate; Peak 4: Catechol; Peak 5: (-) Epicatechin; Peak 6: Caffeic acid; Peak 7: Vanillic acid; Peak 8: Syringic acid; Peak 9: Rutin hydrate; Peak 10: p-Coumaric acid; Peak 11: Trans-Ferulic acid; Peak 12: Rosmarinic acid; Peak 13: Myricetin; Peak 14: Quercetin; Peak 15: Trans-Cinnamic acid; Peak 16: Kaempferol.

**Fig. 2:**
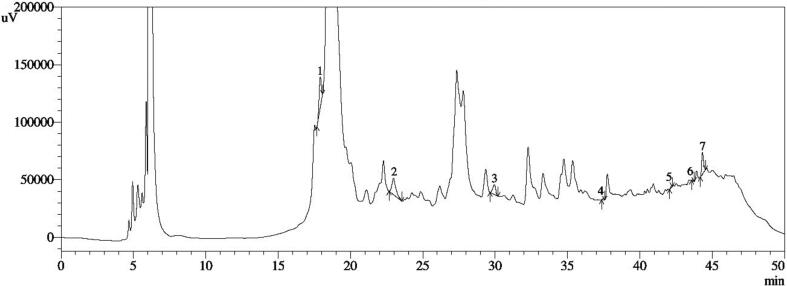
HPLC fingerprint of *C. quinquefolia* (1. 3, 4 dihyrdoxybenzoic acid; 2. Catechol; 3. Rutin hydrate; 4. Myricetin; 5. Quercetin; 6. Trans-Cinnamic acid; 7. Kaempferol.

**Fig. 3:**
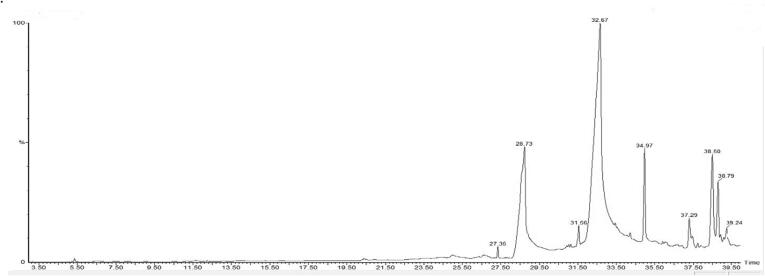
GCMS chromatogram of ethanol extract of *C. quinquefolia.*

The total phenolic content of the extract was found to be 80.6 ± 2.3 mg GAE/g of dry extract from the standard gallic acid calibration curve. While, from the quercetin calibration curve, the total flavonoid content of the extract was calculated as 508.05 ± 3.1 mg QE/g dry extract. The total terpenoid content of the extract was measured as 746.5 ± 2.1 mg ursolic acid/g dry extract. Additionally, HPLC analysis was executed to estimate the polyphenols present in the sample. The chromatographic separations and identification of the polyphenols in standard and extract have been shown in Fig. 1 and Fig. 2, respectively. The findings showed that the catechol content in *C. quinquefolia* was high (69.75 ± 0.51 mg/100 g dry extract), whereas the amounts of 3, 4 dihydroxybenzoic acid, rutin hydrate, and kaempferol were moderate (30.22 ± 0.49 mg/100 g, 25.76 ± 0.04 mg/100 g, 13.70 ± 0.11 mg/100 g dry extract, respectively). Moreover, lesser quantities of myricetin, quercetin, and *trans*-cinnamic acid (1.380 ± 05, 1.240 ± 03, and 0.250 ± 02 mg/100 g of dry extract, correspondingly) were also detected. A GC–MS test was used to investigate the unknown constituents of the plant extract. The chromatogram of GC- MS analysis of the plant is displayed in Fig. 3.

The GC–MS findings revealed the composition of about 16 phytochemicals with their retention time, molecular weight, and area (%) that are given in Table 1. According to the chromatograph, the identified compounds were 1-prophyl 11, 12-methane-octadecanoate (56.17 %), and n-hexadecanoic acid (18.98 %). It also indicated that the moderate number of compounds was glycidyl oleate (5.34 %), glycidyl palmitate (4.32 %), n-propyl 9, cis, 11, trans, - octadecadienoate (2.44 %), 12, 3, 4-tetraydroxy-3-(phenylacetatamido) (2.12 %), and the remaining compounds were less than 2 %.

**Table 1:** Phytochemical composition of *C. quinquefolia* extract through GCMS.

Serial No	Retention Time	Compounds	Molecular weight	Molecular Formula	%Area
1	5.321	Tetraethyl silicate	208	C_8_H_20_O_4_Si	0.15
2	8.100	Furan, 2-methoxy	98	C_5_H_6_O_2_	0.05
3	11.679	1-butanol, 3-methyl formate	116	C_6_H_12_O_2_	0.12
4	12.242	(2E, 2E)-ethane-1,2-diyl bis (2-methylbut-2-enoate)	226	C_12_H_18_O_4_	0.06
5	20.390	Z-23-dotriaconten-2-one	462	C_32_H_62_O	0.23
6	25.001	Orcinol, 2TMS derivative	268	C_13_H_24_O_2_Si_2_	1.18
7	27	Tetradecanoic acid, 10, 13-dimethyl-, methyl ester	270	C_17_H_34_O_2_	0.05
8	27.348	Methyl 11-methyl-dodecanoate	228	C_14_H_28_O_2_	0.43
9	28.735	N-hexadecanoic acid	256	C_16_H_32_O_2_	18.98
10	31.556	11, 14, 17-eicosatrinoic acid, methyl ester	320	C_21_H_36_O_2_	1.09
11	32.669	1-prophy 11, 12-methane-octadecanoate	338	C_22_H_42_O_2_	56.17
12	34.974	Glycidyl palmtate	312	C_19_H_36_O_3_	4.32
13	37.300	12,3,4-tetrahydroxy-3-(phenylacetatamido) quinoline	266	C17H18ON2	2.13
14	38.500	Glycidyl oleate	338	C_21_H_38_O_3_	5.34
15	38.795	N-propyl 9, cis, 11, trans, - octadecadienoate	322	C_21_H_38_O_2_	2.44
16	39.237	Silane, dimethyl (dimethyl(but-3-enyloxy) siloxy) tridecyloxy	402	C_21_H_46_O_3_Si_2_	0.98

### 3.2 Analysis of antioxidant activity

The extract had an IC_50_ value of 1244.5 ± 0.1 μg/mL in the DPPH radical scavenging experiment, while conventional ascorbic acid had an IC_50_ of 19.1 ± 0.13 μg/mL. Also, the ferric reducing antioxidant power of the extract was found to be 40 ± 2.8 mg L-ascorbic acid equivalent per gram of dry extract. Besides, the reducing power of the extract was found to be concentration dependent. The maximum absorbance of 0.016 ± 0.01 was observed at the highest concentration of the extract. Ascorbic acid, used as the positive control, showed a maximum absorbance of 0.25 ± 0.1 at the same concentration. At concentrations of 20, 40, 60, and 80 µg/mL extract showed absorbances of 0.007 ± 0.01, 0.01 ± 0.03, and 0.012 ± 0.0 while ascorbic acid showed absorbances of 0.04 ± 0.0, 0.09 ± 0.03, 0.11 ± 0.04, and 0.15 ± 0.05, respectively.

### 3.3 *In vitro* anti-diabetic activity test

In α-amylase and α-glucosidase inhibitory activity assays, the IC_50_ values of extract were 12.8 ± 0.1 µg/mL and 36.3 ± 0.07 μg/mL, respectively whereas standard acarbose showed IC_50_ values of 7.6 ± 0.1 µg/mL and of 53.6 ± 0.08 μg/mL, respectively.

### 3.4 *In vivo* anti-diabetic activity assay

#### 3.4.1 Oral glucose tolerance test

The anti-diabetic effect of the extract in the oral glucose tolerance test (OGTT) in non-diabetic mice is displayed in Table 2. Blood glucose levels were drastically lowered by treatment with dose of 200 and 400 mg/kg by 1.1 % and 18.9 % at 30 min, respectively, and a notable decline was seen in the hours that followed. After 30, 60, 90, and 120 min after glucose delivery, mice treated with the standard oral anti-diabetic medication glibenclamide significantly (p < 0.05) lower blood glucose levels than the control group (Table 2).

**Table 2:** Effect of extract on mice during oral glucose tolerance test.

Treatment	Blood glucose level a(mmol/L) (% reduction of blood glucose level)				
	0 min	30 min	60 min	90 min	120 min
Control (10 mL/kg)	10.14 ± 1.2	20.14 ± 2.5	17.74 ± 2.1	14.52 ± 1.3	11.62 ± 2.6
Glibenclamide (5 mg/kg)	9.54 ± 1.3 (5.9 %)	13.2 ± 3* (34.5 %)	9.54 ± 2.5* (46.2 %)	5.4 ± 1.9* (62.8 %)	5.26 ± 0.7* (54.7 %)
Extract (200 mg/kg)	10.06 ± 1.1 (0.8 %)	19.9 ± 2.2* (1.2 %)	17.66 ± 2.8* (2.2 %)	14.04 ± 2.5* (3.3 %)	11.22 ± 1.4* (3.5 %)
Extract (400 mg/kg)	9.14 ± 1.8 (9.9 %)	16.32 ± 2.4* (18.9 %)	13.76 ± 2* (22.4 %)	9.1 ± 2.3* (37.3 %)	9.4 ± 2* (19.1 %)

a Values represent as mean ± SD, n = 5, *p < 0.05 vs. control, Student’s *t*-test.

#### 3.4.2 Diabetogenic effect of streptozotocin in mice

The outcomes of the available investigation effectively disclosed that the extract revealed noteworthy hypoglycemic action (P < 0.05) in STZ-diabetic mice, comparable to the effect manifested by standard drug glibenclamide (Table 3), which was caused by diminished insulin levels prompted through streptozotocin-induced deterioration of pancreatic b-cells. On the 21st day, maximum glucose reduction was observed 11.67 % with dose of 200 mg/kg compared to the control. When compared to diabetic control, glibenclamide and extract (400 mg/kg) reduced blood glucose levels by 1.3 % and 16.7 %, respectively, at the end of the trial. According to the findings, the extract could be responsible for the increase of insulin release. Furthermore, the extract's reported reduced ability to lower blood sugar might potentially be related to a rise in peripheral glucose uptake.

**Table 3:** Effect of ethanol extract on fasting blood glucose level in diabetic mice.

Treatment	Blood glucose level a(mmol/L)				
	Day 1	Day 7	Day 11	Day 15	Day 21
Control (10 mL/kg)	7.7 ± 1.1	8.1 ± 0.8	8.6 ± 0.8	8.7 ± 0.7	7.9 ± 0.9
Diabetic Control	10.1 ± 1.6	9.1 ± 0.6	8.8 ± 1.0	9 ± 0.9	7.8 ± 0.7
Glibenclamide (5 mg/kg)	9.4 ± 0.7	7.7 ± 1.1*	7.7 ± 1.1*	7.7 ± 1.1*	7.7 ± 1.1*
Extract (200 mg/kg)	10.7 ± 0.4	8 ± 0.7**	8.7 ± 0.7**	8.1 ± 0.9**	6.8 ± 1.3**
Extract (400 mg/kg)	9.6 ± 0.7	7.1 ± 1.2*	7.6 ± 0.3*	7.9 ± 0.5*	6.5 ± 0.8*

a Values represent as mean ± SD, n = 5, *p < 0.05, **p < 0.09 vs. control, Student’s *t*-test.

### 3.5 Toxicity assay

The LC_50_ values obtained from brine shrimp lethality bioassay for *C. quinquefolia* L. and standard vincristine sulphate were 77.158 ± 0.05 μg/mL and 1.06 ± 0.02 μg/mL, respectively. When the extract was orally administered at dosages of 1000, 2000, and 4000 mg/kg body weight, there were no overt indicators of acute toxicity and no deaths over a period of 14 days. Thus, it was found that the extract's mortality levels (LD_50_) are just more than 4000 mg/kg body weight.

### 3.6 Findings of *in silico* analysis

#### 3.6.1 Visualization of molecular docking and post-docking

**Fig. 4:**
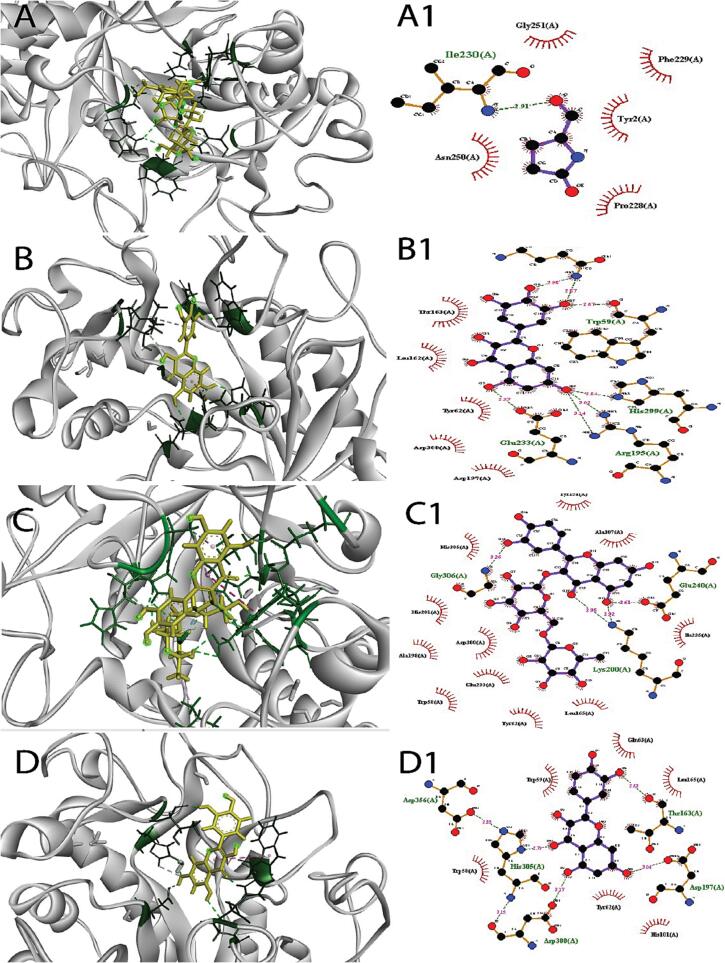
Binding of compounds with IB2Y. Left sides in figure represent the 3D structure and the right side indicates a 2D structure of ligand–protein binding complexes. In 3D structure, ligand molecules were represented in golden colour with adjacent amino acid residues of IB2Y. Right side in figure showed hydrogen bond in olive dotted green color. Here, (**A-A**) was control drug Acarbose. Other compounds were (**B–B**) Myricetin, (**C–C)** Rutin, (**D-D**) Quercetin. (For interpretation of the references to colour in this figure legend, the reader is referred to the Web version of this article). (For interpretation of the references to color in this figure legend, the reader is referred to the web version of this article.)

To estimate the pharmacokinetic properties of the 23 compounds, ADMET analysis was done (Table 3). IB2Y, 3WY1, 6JB3, and 8B8Z proteins were utilized to find phytochemicals that interact with them using a molecular docking approach on the Maestro package platform. In this analysis, acarbose, miglitol, and repaglinide, were used as control. However, the ligand binding affinity to the targeted proteins has presented in table 4, 5, 6, and 7. The interactions between the ligands to distinct proteins were examined using the BIOVIA Discovery Studio Visualizer and Ligplot Plus Version 2.2 tools. For all of the docked complexes, Ligplot + version 2.2 was used to compute the interactions, which were primarily hydrophobic and noncovalent, as indicated in Table 4, Table 5, Table 6, Table 7 and Fig. 4 and Figure S1-S3.(See Table 8).

**Table 4:** The pharmacokinetic and pharmacophore analysis of selected phytochemicals.

**Ligand name**		**MW**	**NHA**	**NHD**	**Log P**	**NRB**	**IA**	**TC**	**BBB**	**LD 50**	**HT**	**AT**	**MTD**	**NLV**	**DL**
Tetraethyl silicate		208.33	4	0	1.22	8	96.412	1.121	Y	2.397	N	N	0.795	0	Y
Furan, 2-methoxy		98.1	2	0	1.18	1	100	0.674	Y	2.492	N	N	1.111	0	Y
1-butanol, 3-methyl formate		116.16	2	0	1.46	4	100	0.24	Y	1.747	N	N	0.949	0	Y
(2E, 2′E)-ethane-1,2-diyl bis (2-methylbut-2-enoate)		226.27	4	0	2.34	5	97.054	1.086	Y	2.072	N	N	0.783	0	Y
Z-23-dotriaconten-2-one		462.84	1	0	10.89	28	87.296	2.105	N	1.914	N	N	−0.306	1	Y
Orcinol, 2TMS derivative		268.50	2	0	3.46	4	91.625	0.936	Y	2.486	N	N	0.55	0	Y
Tetradecanoic acid, 10, 13-dimethyl-, methyl ester		270.45	2	0	5.40	12	100	1.632	Y	1.609	N	N	0.277	1	Y
Methyl 11-methyl-dodecanoate		228.37	2	0	4.40	10	93.511	1.607	Y	1.6	N	N	0.303	0	Y
N-hexadecanoic acid		256.43	1	1	5.20	14	92.004	1.763	Y	1.44	N	N	−0.708	1	Y
11, 14, 17-eicosatrinoic acid, methyl ester		320.51	1	1	6.11	15	91.041	2.033	N	1.457	N	N	−0.812	1	Y
1-prophyl 11, 12-methane-octadecanoate		338.57	2	0	6.86	16	92.1	1.612	N	1.647	N	N	−0.093	1	Y
Glycidyl palmtate		312.49	3	0	5.52	16	93.284	1.719	Y	1.633	N	N	0.125	0	Y
12,3,4-tetrahydro-3-(phenylacetatamido) quinoline		266.34	2	2	2.47	3	93.211	0.273	Y	2.25	N	N	−0.304	0	Y
Glycidyl oleate		338.53	3	0	5.89	17	93.103	1.829	N	1.672	N	N	0.025	0	Y
N-propyl 9, cis, 11, trans, - octadecadienoate		322.53	2	0	6.54	16	92.135	2.088	N	1.588	N	N	0.102	1	Y
Silane, dimethyl (dimethyl(but-3-enyloxy) siloxy) tridecyloxy		402.76	3	0	6.18	19	89.043	1.776	N	2.227	N	N	0.329	0	Y
3,4 dihyrdoxybenzoic acid		154.12	3	3	0.65	1	71.174	0.551	N	2.423	N	N	0.814	0	Y
Catechol		110.11	2	2	0.97	0	86.856	0.147	Y	2.14	N	N	−0.017	0	Y
Rutin hydrate		628.53	16	10	−1.52	6	18.536	0.332	N	2.491	N	N	0.451	3	N
Myricetin		318.23	8	6	0.79	1	65.93	0.422	N	2.497	N	N	0.51	1	Y
Quercetin		302.23	7	5	1.23	1	77.207	0.407	N	2.471	N	N	0.499	0	Y
Trans-Cinnamic acid		148.16	1	1	1.79	2	94.833	0.781	Y	2.094	N	N	1.11	0	Y
Kaempferol		286.23	6	4	1.58	1	74.29	0.477	N	2.449	N	N	0.531	0	Y

**MW**-molecular weight (g/mol); **NHA-** No. of hydrogen bond acceptor; **NHD-** No. of hydrogen bond donor; L**ogP-**Predicted octanol/water partition coefficient; **NRB-** No. of rotatable bonds. **IA**-Intestinal absorption (% absorbed); **TC-**Total clearance (log mL/min/kg); **BBB-** Blood Brain Barrier; **LD50-** Oral rat acute toxicity; **HT-** Hepatotoxicity; **AT-** AMES toxicity; **MTD-** Maximum tolerated dose for human (log mg/kg/day); **NLV-** Number of Lipinski’s Violation; **DL-** Drug Likeness; Y: Yes; N: No.

**Table 5:** Binding affinity of ligands with targeted protein 1B2Y and comprehensive intermolecular interaction.

**Ligand Name (PubChem CID)**	**Binding Affinity (Kcal/mol)**	**Amino Acid Involved Interaction**	
		**Hydrogen Bond**	**Hydrophobic Bond**
Acarbose (41774)	−12.43	Ile230 (2.91 Å)	Gly251(A), Phe229(A), Try2(A), Pro228(A), Asn250(A)
Myricetin (521672)	−9.44	Gln63(2.98 Å), Gln63(2.87 Å), Trp59(2.67 Å), His299(2.84 Å), Arg195(3.02 Å), Arg195(3.14 Å), Glu233(2.97 Å)	Thr163(A), Leu162(A), Tyr62(A), Asp300(A), Asp197(A)
Rutin (5280805)	−9.24	Gly306(3.26 Å) Gln240(2.61 Å), Lys200(2.95 Å), Lys200(2.92 Å)	Tyr151(A), Ala307(A), Ile235(A), Leu165(A), Tyr62(A), Trp58(A), Glu233(A), Asp300(A), His201(A), His305(A)
Quercetin (5280343)	−6.96	Asp356(2.85 Å), is305(2.79 Å), Asp300(3.15 Å), Asp300(3.17 Å, Asp197(3.04 Å), Thr163(2.63 Å)	Trp59(A), Glu63(A), Leu165(A), Tyr62(A), His101(A), Trp58(A)
Kaempferol (5280863)	−5.32	His201(2.90 Å), Glu233(3.05 Å)	Ala198(A), Ile235(A), Tyr151(A), Asp300(A), Trp59(A), Trp58(A), Tyr62(A), Asp197(A)
3,4 dihyrdoxybenzoic acid (72)	−4.61	Tyr62(2.89 Å), Asp197(2.55 Å)	Leu165(A), Gln63(A), His101(A)
11, 14, 17-eicosatrinoic acid, methyl ester (12381801)	−4.29	Ser112(2.78 Å), Tyr52(3.07 Å)	His305(A), Gln63(A), Leu165(A), Trp59(A), Thr163(A), Ala106(A), Ser108(A), Ala50(A), Ile51(A)
12,3,4-tetrahydro-3-(phenylacetatamido) quinolone (582648)	−4.04	Asp197(2.94 Å)	Trp58(A), His299(A), Tyr62(A), Asp300(A), His201(A), Glu233(A), Ile235(A), Leu162(A), Typ151(A), Gly306(A), Lys200(A)
N-propyl 9, cis, 11, trans, - octadecadienoate (91694368)	−3.86	Tyr52(A)	Tyr62(A), Trp58(A), Leu165(A), Trp59(A), Gln63(A), Ile51(A), Val49(A), Ser112(A), Val107(A), Ala50(A), Ser108(A)
Trans-Cinnamic acid (444539)	−3.84	Gln63(3.09 Å)	Leu165(A), Trp59(A), Tyr62(A), Asp300(A), Asp197(A), His299(A)

**Table 6:** Binding affinity of ligands with targeted 3WY1 and comprehensive intermolecular interaction.

**Ligand Name (PubChem CID)**	**Binding Affinity (Kcal/mol)**	**Amino Acid Involved Interaction**	
		**Hydrogen Bond**	**Hydrophobic Bond**
Miglitol Control (441314)	−6.08	Arg200(2.96 Å), Asp202(2.95 Å), Gly228(2.85 Å), Gly228(2.71 Å)	Glu271(A), Thr203(A), Ile146(A), Tyr389, Arg400(A), Asp333(A), Tyr65(A)
12,3,4-tetrahydro-3(phenylacetalatamido) quinolone (582648)	−8.01	Gly228(2.98 Å)	Ala229(A), Phe397(A), Val334(A), Arg400(A), Pro230(A), Asp333(A), Ile146(A), Leu227(A), Tyr389(A), Phe297(A), Phe206(A), Glu271(A), Gly273(A)
Myricetin (521672)	−7.63	Glu271(2.71 Å), Asp333(3.06 Å), Asn301(3.07 Å), Leu227(2.99 Å)	Thr203(A), Tyr389(A), Arg400(A), Phe397(A), Val334(A), Gly228(A), Ala229(A), Phe297(A), Gly273(A)
Quercetin (5280343)	−7.18	Arg400(2.66 Å), Asp333(2.82 Å), Leu227(2.75 Å), Asn301(A)	Tyr389(A), Phe166(A), Gly273(A), Phe297(A), Gly228(A), Val334(A), Phe397(A), Pro230(A)
3,4 dihydrobenzoic acid (72)	−5.79	Glu271(2.71 Å), Arg200(2.92 Å), Asp333(3.04 Å), Gln170(3.08 Å), Asp62(2.52 Å)	Thr203(A), Phe166(A), Asp202(A), Tyr65(A), Arg400(A)
Kaempferol (5280863)	−5.34	His105(3.25 Å), Asp202(3.06 Å), Asp333(2.63 Å), Arg400(2.72 Å)	Tyr235(A), Ile146(A), Phe206(A), Gly273(A), Gly228(A), Thr203(A), Phe297(A), Phe166(A), Tyr65(A), His332(A)
Trans-Cinna-mic acid (444539)	−4.71	Arg200(2.99 Å), Arg200(2.96 Å)	Gly228(A), Glu271(A), Asp333(A), His332(A), Asp202(A), Tyr65(A)
Glycidyl palmtate (347736)	−4.35	Asp274(2.75 Å), Thr226(2.74 Å)	Ile272(A), Pro277(A), Phe297(A), Gly273(A), Ile176(A), Tyr235(A), Thr203(A), Phe206(A), Gly228(A), Glu271(A), Asp333(A), Tyr389(A), Arg400(A)
Orcinol,2TMS derivative (522920)	−4.14	Arg400(2.95 Å), Tyr389(3.30 Å)	Asp202(A), Arg200(A), Phe166(A), Glu271(A), Phe206(A), Tyr65(A), Ile146(A), Phe297(A), Asp333(A)
N-hexadec-anoic acid (985)	−4.05	Leu227(2.73 Å)	Phe206(A), Thr226(A), Tyr235(A), Asp274(A), Asn301(A), Gly273(A), Gly228(A), Asp333(A), Tyr389(A), Phe397(A), Pro230(A), Ala229(A)

**Table 7:** Binding affinity of ligands with targeted 6JB3 and comprehensive intermolecular interaction.

Ligand Name (PubChem CID)	Binding Affinity (Kcal/mol)	Amino Acid Involved Interaction	
		Hydrogen Bond	Hydrophobic Bond
Repaglinide (65981)	−6.56	Trp12979(2.91 Å),	Met1290(B), His584(B), Thr588(B), Asn1293(B), Asn547(B), Thr548(B), Arg1145(B), Phe591(B), Cys1142(B)
Rutin (5280805)	−12.57	Asn1293(3.09 Å), Glu1253(2.62 Å), Asn1296(2.97 Å), Tyr377 (2.91 Å), Arg1300(2.82 Å)	Asn547(B), Tyr548(B), Val667(B), Arg1145(B), Leu1149(B), Phe591(B), Met1290(B), Trp1297(B), Tyr129(B), His584(B), Thr566(B), Gln369(B), Leu373(B), Leu592(B)
Quercetin (5280343)	−9.1	Asn547(2.89 Å), Asn1293 (2.90 Å), Tyr1294(2.95 Å)	Leu1149(B), Thy548(B), Val587(B), Phe591(B), Leu592(B), Thr588(B), Trp1297(B)
Kaempferol (5280863)	−8.09	Asn547(2.84 Å), Asn1293(2.87 Å)	Thr548(B), Arg1145(B), Leu1149(B), Phe591(B), Thr588(B), Leu592(B), Trp1297(B)
Myricetin (521672)	−7.7	Asp310(2.72 Å), Gln444(2.79 Å), Arg306(2.96 Å), Asn1296 (3.06 Å), Asn1296(3.05 Å), Arg1300(3.05 Å), Gln (2.79 Å)	Ile585(B), Leu373(B), Thr588(B), Tyr377(B)
11, 14, 17-eicosatrinoic acid, methyl ester (123481801)	−6.85	Gln369(2.70 Å), Arg370(2.77 Å), Arg370(2.64 Å)	Trp1297(B), Phe591(B), Leu592(B), Tyr377(B), Leu373(B), Ile585(B), Asn1293(B), Val1587(B), Thr588(B), Asn547(B)
12,3,4-tetrahydro-3-(phenylacetatamido) quinolone (582648)	−5.96	–	Arg1145(B), Asn547(B), Thr548(B), Thr588(B), Asn1293(B), Trp1297(B), Leu1149(B)
Z-23-dotriaconten-2-one (21159942)	−5.48	Asn1245(2.93 Å)	Gln1134(B), Ser1138(B), Ile544(B), Cys1142(B), Trp1297(B), Leu592(B), Tyr377(B), Asn437(B), Arg1246(B), Ile381(B), Thr1242(B), Trp430(B), Phe591(B), Arg1145(B), Ser543(B)
Glycidyl oleate (5354568)	−5.31	Asn1296(3.09 Å), Tyr377(3.15 Å)	Thr1242(B), Leu1241(B), Ile381(B), Trp430(B), Leu434(B), Glu1249(B), Asn1293(B), Glu1253(B), Arg1300(B), Leu373(B), Arg1245(B), Phe433(B), Ser1238(B)
1-prophyl 11, 12-methane-octadecanoate (91692516)	−5.27	Thr588(3.15 Å)	Phe433(B), Ile381(B0, Arg1246(B), Leu434(B0, Trp430(B), Trp1297(B0, Asn1293(B), Tyr377(B), Leu592(B), Asn437(B)

**Table 8:** Binding affinity of ligands with targeted 8B8Z and comprehensive intermolecular interaction.

Ligand Name (PubChem CID)	Binding Affinity (Kcal/mol)	Amino Acid Involved Interaction	
		Hydrogen Bond	Hydrophobic Bond
Repaglinide Control (65981)	−8.89	Lys265(Å)	Ser342(A), His266(A), Phe264(A), Gly284(A), Ile341(A), LLeu333(A), Ser289(A), Ala285(A), Leu330(A), Leu340(A), Arg288(A), Met364(A), Glu291(A), Phe363(A)
Rutin (5280805)	−14.52	Ser289(Å)	Phe264(A), His266(A), Lys265(A), Phe287(A), Ser342(A), Met329(A), Arg288(A), Leu333(A), Ala292(A), Ile326(A), His449(A), Leu330(A), Met364(A), Phe363(A), Ala285(A), Met348(A), Ile281(A), Gly284(A), Ile341(A)
Z-23-dotriaconten-2-one (21159942)	−10.07	Arg288(2.84 Å)	Leu228(A), Glu295(A), Leu333(A), Met329(A), Ile326(A), Ala292(A), Leu330(A), Ile341(A), Met364(A), Ile281(A), Glu259(A), Met348(A), Phe264(A), Arg280(A), His266(A), Phe287(A), Ile267(A), Gly284(A), Ala285(A), Ser289(A), Phe226(A)
Myricetin (5281672)	−9.89	Glu259(2.83 Å) Glu259(3.33 Å)	Arg280(A), Gly284(A), Phe264(A), Arg288(A), Ile341(A), Met364(A), Leu353(A), Met348(A), Ile281(A)
11, 14, 17-eicosatrinoic acid, methyl ester (123481801)	−9.5	Ser342(3.23 Å) Lys265(2.74 Å)	Ile281(A), Ile341(A), Gly284(A), Val339(A), Phe363(A), Met348(A), Leu353(A), Ala285(A), Met364(A), Leu330(A), Ser289(A), Ala292(A), Arg288(A), Phe287(A), Phe264(A)
Quercetin (5280343)	−9.28	Glu259(2.83 Å) Glu259(3.33 Å)	Arg280(A), Phe264(A), Gly284(A), Ile341(A), Arg288(A), Leu353(A), Met364(A), Ile281(A), Met348(A)
1-prophyl 11, 12-methane-octadecanoate (91692516)	−9.01	Arg288(2.91 Å)	Phe264(A), Ile341(A), Met364(A), Ser289(A), Phe363(A), Leu333(A0, Phe226(A), Glu295(A), Ile326(A), Met329(A), Ala292(A), Leu330(A), Ala285(A), Met348(A), Glu259(A), Ile281(A)
Glycidyl oleate (5354568)	−8.57	Lys265(2.82 Å)	Glu291(A), Glu343(A), Arg288(A), Ala292(A), Ser289(A), Ile326(A), Ile281(A), Met348(A), Phe363(A), Ala285(A), Ile341(A), Gly284(A), Ser342(A), Phe264(A)
Glycidyl palmtate (347736)	−8.35	Lys265(2.92 Å)	His266(A), Ile267(A), Arg280(A), Ile281(A), Met348(A), Met364(A), Leu330(A), Ile341(A), Ala285(A), Phe264(A), Gly284(A), Phe287(A)
N-propyl 9, cis, 11, trans, - octadecadienoate (91694368)	−8.31	–	Ile281(A), Met364(A), Val339(A), Ser289(A), Ile326(A), Ala285(A), Ala292(A), Leu330(A), Phe226(A), Leu333(A), Glu295(A), Ser332(A), Leu228(A), Thr229(A), Lys230(A), Arg288(A), Met329(A), Ile341(A)

#### 3.6.2 Molecular dynamic simulation

To further explore the structural changes of the protein in complex, a 100 ns MD simulation of the protein in combination with the specific ligand was conducted in this study. This method may also be used to identify conformational changes in complex systems that have been exposed to a controlled environment. The terminal snapshots from the MDS trajectory were first used to study the intermolecular behaviour (Aljahdali et al., 2021).

##### 3.6.2.1 RMSD analysis

**Fig. 5:**
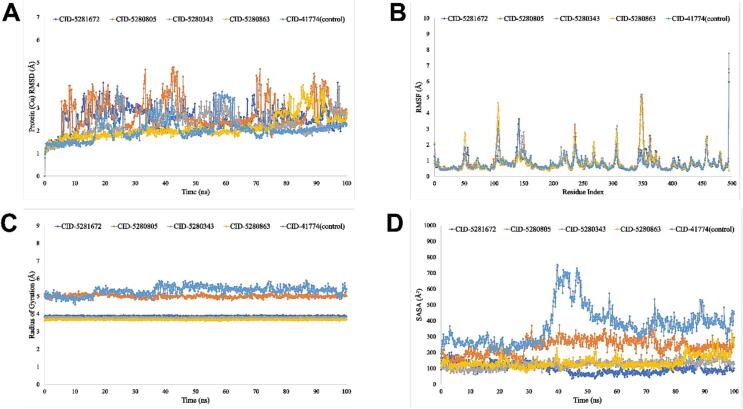
The RMSD, RMSF, Rg and SASA values for 1B2Y in complex with the five ligand compounds recovered from the complex system’s C atoms. Where blue, orange, grey, yellow and sky-blue colour represent the selected five ligand compounds. (For interpretation of the references to color in this figure legend, the reader is referred to the web version of this article.)

The stability of the ligands and the control ligand was assessed using individual plots of the RMSD (Root Mean Square Deviation) graph. The average change in the RMSD value is suitable with a range of 1–3 Å. The protein structure has altered considerably if the RMSD value is more than 1–3 Å. The average RMSD value for 1B2Y-ligand compounds CID-5280343 and CID-5280863 was 1–3 Å, whereas CID-41774 (control) was 1–3.5 Å (Fig. 5). The protein–ligand complex structure shown in Figure S5–S7 has very minimal variation for the 3WY1, 6JB3, and 8B8Z proteins, which suggest the constant conformation of the proteins.

##### 3.6.2.2 RMSF analysis

When ligands were enmeshed in the protein binding site, the adaptability of the protein–ligand complexes was investigated. To analyse the dynamic variations of the protein–ligand complex with respect to time, the Root Mean Square Fluctuation (RMSF) was employed. The RMSF value is studied in order to understand the characteristics of proteins that reveal information about the real protein structure. After examining Fig. 5 and Figure S5–S7, it was determined that the best inhibitors are myricetin (PubChem CID: 521672), rutin (PubChem CID: 5280805), and Z-23-dotriaconten-2-one (PubChem CID: 21159942).

##### 3.6.2.3 The radius of gyration (Rg) analysis

A protein–ligand interaction system's radius of gyration (Rg) is determined by the configuration of its atoms along its axis. Since it reveals changes in complex compactness over time, the computation of Rg is one of the most crucial signs to look for when predicting structural functioning of a macromolecule. The average Rg values for the compounds’ in IB2Y PubChem CIDs 5281672, 5280805, 5280343, 5280863, and 41,774 (control) were 3.8, 5, 3.7, 3.6, and 5.5 Å, respectively (Fig. 5 and Figure S5–S7). The proteins 3WY1, 6JB3, and 8B8Z show that when the ligand molecules are bound during the course of a 100 ns simulation, the protein's binding site does not significantly alter structurally.

##### 3.6.2.4 Analysis of SASA, MolSA, and PSA

The solvent-accessible surface area (SASA) of the simulated trajectories has been examined to provide an explanation for the variations in the protein surface region. The SASA values for IB2Y protein of four compounds (CID 5281672, CID 5280805, CID 5280343, and CID 5280863) were 100 to 300 Å^2^ on average, and 700 Å^2^ for CID 41774. For 3WY1, 6JB3, and 8B8Z proteins, the SASA value was 0–160 Å^2^ on average. The MolSA surface calculation model has a 1.4 Å probe radius. An area of a Vander Waals surface is represented by this value. For 1B2Y, 3WY1, 6JB3, and 8B8Z, its value is constant in the range of 250–500 Å^2^, 150–275 Å^2^, 250–480 Å^2^, and 280–600 Å^2^, with minimum fluctuations during the course of the experiment (Fig. 5 and Figure S4–S7). The solvent-accessible surface area for the 1B2Y, 3WY1, 6JB3, and 8B8Z proteins is observed in the range of 100–700 Å^2^, 0–100 Å^2^, 10–200 Å^2^ and 280–600 Å^2^ with minor fluctuations throughout the simulation period (Fig. 5 and S5–S7). The polar solvent-accessible area in the molecule is furnished solely by nitrogen and oxygen atoms. For 1B2Y, 3WY1, 6JB3, and 8B8Z proteins, their range is found from 240 to 550 Å^2^ 240–550 Å^2^, 60–325 Å^2^ 60–325 Å^2^, 100–450 Å^2^ and 0–450 Å^2^ with minor fluctuations in the simulation period (Fig. 5 and Figure S4-S7).

##### 3.6.2.5 Protein-ligand interaction

**Fig. 6:**
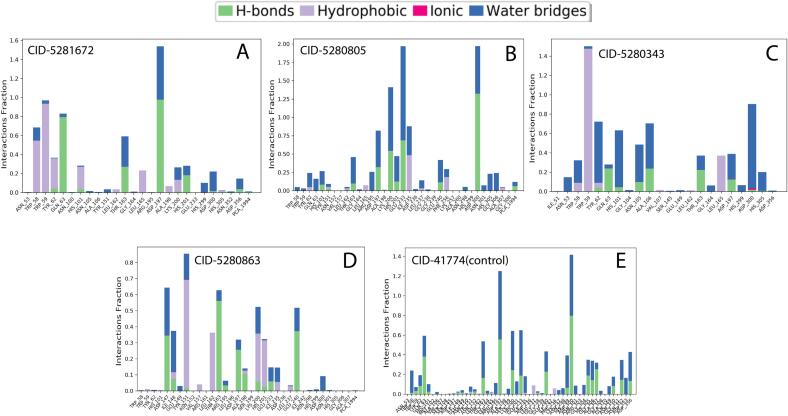
Representation of the 1B2Y protein–ligand interaction by 100 ns MD simulation.

The complicated structure of a protein with the chosen ligands and their intermolecular interactions were examined for 100 ns of simulation duration (SID) using the simulation interactions diagram. The hydrogen bond, the ionic bond, the water bridge bond, and the noncovalent interactions bond (hydrophobic bond) outline the interaction among the 1B2Y, 3WY1, 6JB3, and 8B8Z proteins and the precise ligands. The greatest interaction fraction values for the 1B2Y compound's control, CID 5181672, CID 5280805, CID 5380343, and CID 5280863 are, respectively, 1.4, 1.6, 2, 1.4, and 0.8 at the residues ASP300, ASP197, GLU2333, TRP59, and TYR151 (Fig. 6). The maximum interaction percentage values are above 1, 2, 1, 2, and 1.75 at the residues, ASP202, ASP62, and GLU271 correspondingly in the 3WY1 protein and ligands bar graph, which was demonstrated by the control, CID-528048, CID-5281672, CID-5280343, and CID-72 (Figure S8). The greatest contact fraction values for the 6JB3 protein and its ligands were 1.75, 1.2, 0.8, 1.75, and 1.6 at the residues ASN1293, ASN1293, ASN547, and ASP310, respectively (Figure S9). The fraction values for the 8B8Z protein and its ligands were 0.45, 1.2, 1.6, 2, and 2 at the residues PHE264, SER342, HIS266, GLU259, and Ser342, respectively (Figure S10). All compounds made a variety of linkages during the course of the 100 ns simulation time and maintained these contacts throughout the simulation, thereby facilitating a stable binding with the required protein. These connections were built by hydrogen, ionic, water bridge, and hydrophobic bonding.

#### 3.6.3 Post simulation binding energy calculation (MM/GBSA)

**Fig. 7:**
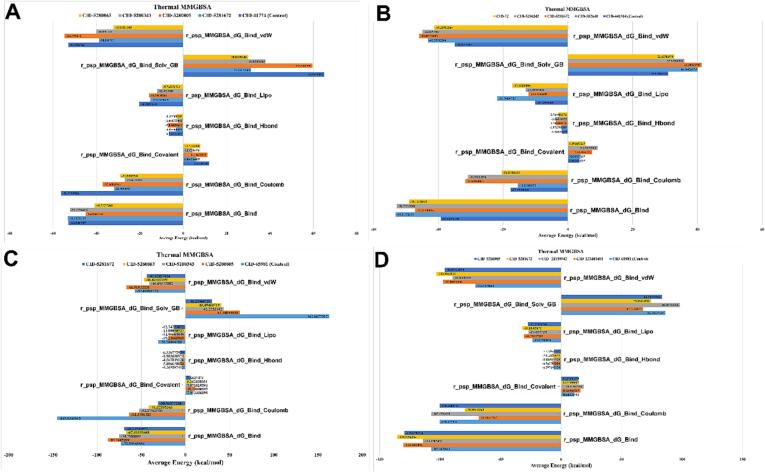
Analysis of post simulation trajectory MM/GBSA of the protein 1B2Y(A), 3WY1(B), 6JB3(C) and 8B8Z(D) with the selected ligand complex.

The molecular mechanics-generalized bound state analysis, or for a group of ligands and a single receptor, born surface area may be utilized to determine the ligand binding free energies and ligand strain energies. Here, the interaction between the ligand molecules and the targeted protein complex is stronger the higher the negative free energies of binding (MMGBSA_dG_Bind_vdW for complex-receptor-ligand) are (Fig. 7). For 1B2Y, the findings strongly depicted that the rutin (CID: 5280805), the acarbose (CID: 41774) (control drug), quercetin (CID: 5280343), and myricetin (CID: 5281672) with the receptor complex exhibited binding free energy of −58, −55, −40, and −39 Kcal/mol, respectively. However, the compound kaempferol (CID: 5280863) showed a lower binding free energy compared to the other compounds at −30 Kcal/mol. The highest and the lowest binding free energies for 3WY1, 6JB3, and 8B8Z are −50, −75, −105, and −35, −40, −75 Kcal/mol, respectively (Fig. 7).

#### 3.6.4 PASS online prediction for QSAR analysis

Using the PASS online tool, all 23 phytochemicals were assessed for their potential to prevent diabetes. The phytochemicals were only taken into consideration based on their combined properties, such as being anti-diabetic, alpha amylase and alpha glucosidase inhibitors. Activities with greater Pa have pharmacological potency and experimental production potential (Samad et al., 2022). In our QSAR model analysis of all the phytocompounds, we have screened the best 23 phytocompounds by utilizing the Pa cut-off value ≥ 100 (greater or equal to 100) (Table 9). Also, PASS can help reduce the side effects of a molecule even though it cannot predict the binding affinity for new therapeutic targets. The 23 filtered phytochemicals mentioned above were taken for site specific molecular docking analysis after completing the ADME evaluation.

**Table 9:** The result of QSAR models for bioactivity prediction in ligand validation.

**PubChem CID**	**Compounds**	**Pa**	**Pi**	**Activity**
6517	Tetraethyl silicate	0.828	0.009	α-glucosidase and α-amylase Inhibitor
		0.459	0.010	Treatment of diabetes
117,476	Furan, 2-methoxy	0.187	0.146	Antidiabetic symptomatic
8052	1-butanol, 3-methyl	0.303	0.030	α-amylase inhibitor
87,104,197	(2E, 2′E)-ethane-1,2-diyl bis (2-methylbut-2-enoate)	0.469	0.008	α-amylase inhibitor
21,159,942	Z-23-dotriaconten-2-one	0.348	0.023	α-amylase inhibitor
		0.131	0.015	α- glucosidase inhibitor
		0.137	0.076	Antidiabetic (type 1)
522,920	Orcinol, 2TMS derivative	0.182	0.066	α-amylase inhibitor
554,145	Tetradecanoic acid, 10, 13-dimethyl-, methyl ester	0.148	0.057	Antidiabetic (type 1)
4,065,233	Methyl 11-methyl-dodecanoate	0.281	0.034	α-amylase inhibitor
		0.164	0.037	Antidiabetic (type 1)
985	N-hexadecanoic acid	0.583	0.003	α-amylase inhibitor
		0.323	0.070	Antidiabetic activity
		0.150	0.010	α-glucosidase inhibitor
123,481,801	11, 14, 17-eicosatrinoic acid, methyl ester	0.369	0.053	Antidiabetic activity
91,692,516	1-prophyl 11, 12-methane-octadecanoate	0.159	0.042	Antidiabetic (type 1)
347,736	Glycidyl palmtate	0.155	0.047	Antidiabetic (type 1)
582,648	12,3,4-tetrahydro-3-(phenyl-acetatamido) quinoline	0.331	0.108	Insulin promoter
5,354,568	Glycidyl oleate	0.368	0.017	Antidiabetic symptomatic
91,694,368	N-propyl 9, cis, 11, trans, - octadecadienoate	0.473	0.008	α-amylase inhibitor
		0.302	0.038	Antidiabetic symptomatic
91,742,954	Silane, dimethyl (dimethyl(but-3-enyloxy) siloxy) tridecyloxy	0.133	0.013	α-glucosidase I inhibitor
289	Catechol	0.473	0.008	α-amylase inhibitor
		0.211	0.014	Antidiabetic (type 1)
45,479,757	Rutin hydrate	0.858	0.001	α-glucosidase inhibitor
		0.528	0.019	Antidiabetic activity
		0.322	0.027	α-amylase inhibitor
5,281,672	Myricetin	0.321	0.003	α-glucosidase inhibitor
5,280,343	Quercetin	0.195	0.019	Antidiabetic (type 1)
444,539	Trans-Cinnamic acid	0.440	0.033	Antidiabetic activity
		0.108	0.025	α-glucosidase inhibitor
5,280,863	Kaempferol	0.196	0.169	Antidiabetic activity
		0.248	0.004	α-glucosidase inhibitor
		0.136	0.103	α-amylase inhibitor

Pa: probable activity; Pi: probable inactivity.

#### 3.6.5 Network pharmacology

**Fig. 8:**
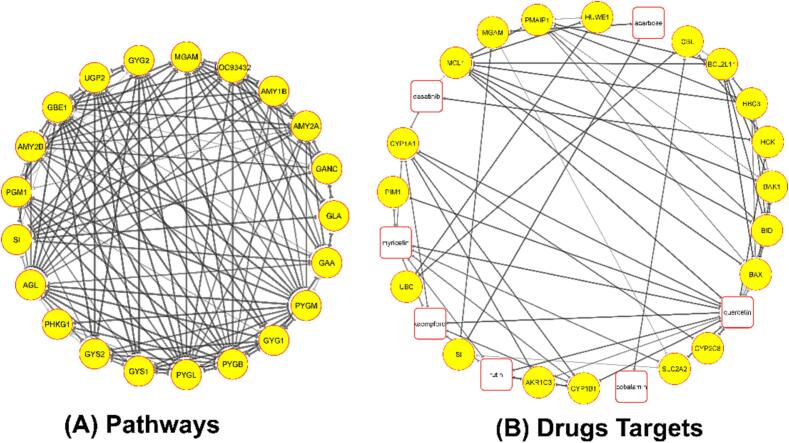
Network Pharmacology analysis of 1B2Y associated diabetes induced genes with the selected drug candidates. Here, (A) Construction of 1B2Y network with the neighbouring genes based on mean interaction sources, (B) Construction of the drug-protein interactions (DPI) using STITCH and Cytoscape mapping to demonstrate multiple diabetes protein targets for the drug candidates.

1B2Y, along with other interactive genes, are represented by nodes, and their in-between interactions are indicated by edges, each having a sender and a receiver side (Fig. 8A). Among the related genes, AMY2A, AMY2B, AMY1B, GANG, GLA, PYGB, GBE1, AGL, PYGL, and PYGB are found to be much more interactive at the edges of the overall gene-gene interaction network with 1B2Y. Most importantly, all the exhibited genes in the string network for 1B2Y are strongly responsible for the induction of diabetes in the human body. The drug-protein circuit network shows the connection between five different drug candidates, with the significant key drugs in white and 19 other diabetes-related protein targets presented here as nodes (Fig. 8B). All the edges show the connection between the drugs and protein targets. Quercetin has both strong edges and most of the nodes, indicating its potential as a drug that can effectively work on numerous diabetes protein targets. In the case of 3WY1, the other interactive genes are shown as nodes, and the interactions that occur between them are shown as edges, each of which has a sender and a recipient side (Figure S11A). Among all related genes, HKDC1, HK1, GP1, AMY1B, AMY2A, NGAM, and OC93432 are exhibited to be much more associated by the edges inside the network of overall gene-gene interaction with 3WY1. In addition, the drug-protein circuit network represented that the drug candidate’s quercetin and myricetin represented significant interactions with 19 diabetes-related protein targets presented here as nodes (Figure S11B). Moreover, in the case of 6JB3, the interactive genes shown as nodes can effectively contribute to the progression of diabetes (Figure 12A). Among all related genes, SNAP25, PRKACA, KCNJ11, STX1A, ABCC8, RAPGEF3, PRKAR1B, PRKACB, RAPGEF4, SYT1, and SNAP23 are shown to be much more correlated by the edges inside the network of overall gene-gene interaction with 6JB3. In addition, the drug candidate’s quercetin, rutin, kaempferol, and myricetin reported significant interactions with 20 diabetes-related protein targets in the drug-protein circuit network (Figure 12B). Furthermore, for the 8B8Z, it represented a potent interaction with the diabetes induction-responsible genes (Figure S13A). Among all related genes, NFKB1, NCOA1, NCOA2, SMAD2, RELA, NR3C1, MED1, CREBBP, NCOR1, NFKBIA, EP300, NCOR2, and SIRT1 are exhibited to be much more associated by the edges inside the network of overall gene-gene interaction with the 8B8Z. In addition, the drug-protein circuit network represented that the drug candidate’s rutin and myricetin represented significant interactions with 20 diabetes-related protein targets presented here as nodes (Figure S13B).

## 4 Discussion

A variety of illnesses are treated with medicinal plants (Rana et al., 2021). *Crotalaria species* are utilized to treat liver illness, stomach aches, and also in diabetes (Yaradua, 2018) due to the presence of bioactive elements. The polyphenol, flavonoids, and terpenoids found in plant products are known to lower blood glucose levels (Sarian et al., 2017). It was discovered that polyphenols and antioxidant properties were positively correlated. The antioxidant potentiality of *C. quinquefolia* was evaluated using three complementary *in vitro* experiments, and the findings showed that the extract exhibited significant antioxidant activity. Antioxidant activities of identified polyphenols of the extract namely catechol, hexadecanoic acid, kaempferol, *trans*-cinnamic acid, quercetin, myricetin, rutin, and 3, 4-dihydroxybenzoic acid have been reported (Agraharam et al., 2022, Smolyaninov et al., 2022). Additionally, polyphenols are known to have strong anti-diabetic properties and to aid in the control of blood glucose level. Different bioactive compounds have been identified in the experimental extract by HPLC and GCMS analysis. Many researchers have examined the relationship between free radicals, antioxidants, and the defence of cellular components vital to the mechanisms of insulin release and glucose absorption. Antioxidants may regulate the release of insulin, the absorption of glucose, and the subsequent anti-hyperglycemic action via a variety of routes. Catechol inhibits insulin receptor signalling, which reduces glucose uptake, downregulates the proteins involved in the insulin signalling pathway, improves glucose metabolism, lowers plasma triglyceride levels, reduces insulin sensitivity, and inhibits Jak-Stat signalling via PPAR γ control (Knezevic et al., 2021). Hexadecanoic acid inhibits α-amylase and α-glucosidase functionality, thereby interfering with the conversion of carbohydrates into glucose (Agada et al., 2021). The flavonoid kaempferol enhances glucose uptake in adipocytes (Fang et al., 2008), lowers blood glucose through inhibiting α-amylase and glucosidase enzymes (Ibitoye et al., 2018), inhibits RhoA/Rho-kinase-mediated inflammatory signalling (Sharma et al., 2019), increases insulin signalling, controls fatty acid metabolism, modulates IR (insulin receptor) to minimize lipotoxicity, reduces glucose toxicity by balancing production and consumption of glucose, and balances autophagy-apoptosis to preserve β-cells (Yang et al., 2022). Trans-cinnamic acid stimulates adiponectin secretion and AMPK activation (Kopp et al., 2014). improving glucose tolerance and stimulating insulin secretion (Hafizur et al., 2015), and preventing obesity by activating the AMPK signalling pathways (Wang et al., 2020). Moreover, Quercetin protects pancreatic damage, encourages restoration of the pancreatic islets, and increases their capacity to sustain normal blood glucose level (Azeem et al., 2023), improving insulin-stimulated glucose absorption in adult adipocytes (Eid et al., 2015). Also, Myricetin acts against diabetes by stimulating lipogenesis in adipocytes and increasing the stimulatory effect of insulin, suppressing the α-glucosidase activity, enhancing the antioxidant defense system, anti-aldose reductase, and insulin resistance (Imran et al., 2021), and protecting the β-cells against HG-induced fatality by preventing ER stress, presumably by the inactivation of CDK5 and subsequent overexpression of PDX1 and SER A2b (Karunakaran et al., 2019). Besides, Rutin improved insulin receptor kinase activity, which helped to support the insulin signaling pathway that boosted glucose transporter and enhanced glucose absorption (Ganeshpurkar & Saluja, 2017), improved insulin secretion and PDX-1 expression and nuclear localization (Lee et al., 2021) and increased adipose tissue PPAR γ expression (Sana et al., 2020). Furthermore, 3,4-dihydroxybenzoic acid increased serum insulin level and β–cell function (Erukainure et al., 2017).

Both enzymatic and non-enzymatic antioxidant defense mechanisms are used to manage diabetics by preventing and/or managing type-2 diabetes as well as limiting the activity of enzymes related to glucose metabolism. By delaying the absorption of carbohydrates after eating, inhibiting α-amylase and α-glucosidase slows down the generation of glucose and finally reduces blood glucose levels (Salahuddin et al., 2020). In the α-amylase and α-glucosidase inhibitory assays, it was noted that the extract IC_50_ values occurred at concentrations of 12.8 ± 0.1 μg/mL and 36.3 ± 0.07 μg/mL respectively, whereas acarbose had an IC_50_ value of 7.6 ± 0.1 μg/mLand 53.6 ± 0.08 μg/mL, respectively. *C. quinquefolia* extract exhibited an IC_50_ value less than that of acarbose, suggesting that it could be a source of a promising antidiabetic lead compound (s). Recently, acarbose has been recognized as a positive control in numerous *in vitro* anti-diabetic experiments due to its powerful digestive enzyme inhibitory activity (Rashid et al., 2022). This work supports the idea that natural blockers derived from plants have amylase inhibitory action and might be employed as a successful treatment for the control of postprandial hyperglycemia with few adverse effects. Using antioxidant substances with antihyperglycemic properties may be the most effective treatment for the illness. The ability of a cell to eliminate glucose after consuming a specified amount of sugar is measured by an oral glucose tolerance test, which may be done by blocking the enzymes that break down carbohydrates, alpha-glucosidase or alpha amylase (Debnath et al., 2020). The plant extract demonstrated a dose-dependent decrease in blood sugar level within the observed time in the oral glucose tolerance test. Over the ensuing hours, the levels of blood sugar were significantly reduced in just the 200 mg/kg treated animals. Different extract treatments (200 and 400 mg/kg) significantly dropped the amount of glucose in the blood by 1.2 % and 18.9 %, respectively, at the end of 30 min. The extract's hypoglycemic impact on diabetic mice generated by streptozotocin are discussed in this article. In glucose-fed hyperglycemic normal mice, the extract at a dosage of 200 mg/kg body weight substantially improved glucose tolerance. This observation could be explained by the stimulation of the insulin effect of plasma, which is accomplished through elevated pancreatic secretion of insulin from functioning b-cells or its release from connected insulin, by a decrease in the rate of gastrointestinal glucose absorption, attained by an extra pancreatic action, such as stimulating the metabolism of peripheral glucose utilization or enhancing glycolytic, improving antioxidant activities and glycogenic process with concomitant decrease in glycogenolysis, and by a surge in the rate of intestinal utilization of glucose (Sharma et al., 2011).

*C quinquefolia* has been shown to have a high inhibitory impact on α-amylase and α-glucosidase, according to the findings of its *in vivo* and *in vitro* anti-diabetic properties. Furthermore, it was claimed to drastically lower the blood glucose levels of mice that were diabetic and loaded with glucose. The necessity for in-silico screening of natural chemicals for drug creation has arisen as a result of the high cost, long screening times, and arduous screening techniques (Abdullah et al., 2023, Arefin et al., 2021, Biswas et al., 2021, Khan et al., 2021, Rahman et al., 2022). Unfocused laboratory processes that lack structural knowledge of medications and target molecules result in waste of time and money. Based on a number of factors, pharmacokinetic assessment evaluates the drug-like properties of substances and compares them to other medications (Olasupo et al., 2020). Based on variables like molecular mass (500 Dalton), molar refractive index (40–130), partition coefficient (LogP ≤ 5), hydrogen bond donors (5), and hydrogen bond acceptors (10), Lipinski's rule of five predicts the drug-like properties or drug potency of suggested compounds**.** Only substances that meet these requirements are regarded as viable drug candidates. Recently, the computational form of drug design approach has acquired widespread popularity for forecasting the effects of noble drug compounds on a variety of diseases using computational techniques. By leveraging computer-simulated environments, researchers can conduct drug discovery and development without the need for costly and time-consuming physical experiments. In molecular docking, a key and lock approach were employed in order to determine complicated protein–ligand complexes having the greatest binding affinities through a sophisticated screening tool Schrödinger Release 2021–2: Maestro, Schrödinger, LLC, New York, NY, 2020–3. Among 23 identified phytochemicals, 4 compounds were chosen for MDS based on the outcomes of the molecular docking. An important technique for analyzing the biomolecular structure of both ligands and proteins is molecular dynamics simulation (MDS), which also gives a notion of how stable the protein–ligand interaction is? Lower RMSD values represent the compounds with the highest stability, while RMSF values assess mean palpitation, which establishes how compact the protein–ligand complex is. Lower Rg values indicate high compactness, while higher values show that the compounds have become disassociated from the protein. A higher SASA value denotes a more unstable structure, while a densely connected combination of water molecules and amino acid residues is indicated by a smaller number (Baildya et al., 2021). In MMGBSA analysis, the low energy of the complexes indicates their stability during the whole simulation time; more negative values indicated better stability. QSAR analysis and network pharmacology established probable antidiabetic activity and predicted gene target pathways. However, among 23 phytochemicals, myricetin (PubChem CID: 521672); rutin (PubChem CID: 5280805); quercetin (PubChem CID: 5280343); and kaempferol (PubChem CID: 5280863) from *C. quinquefolia* showed anti-diabetic activity. Both experimental and computational studies revealed the scientific applications of the experimental plant extract for anti-diabetic disorders. Consequently, a number of the chemicals from the plant may be used as leads for IB2Y, 3WY1, 6JB3, and 8B8Z modulators in future drug-likeness studies.

Therefore, more in-depth research is required to pinpoint the precise mechanism of action of these extracts and to highlight the key bioactive substances relevant for these antidiabetic properties for the creation of novel plant-based medications.

## 5 Conclusions

In the current investigation, *C. quinquefolia* extract exhibited significant anti-diabetic effect in both *in-vivo* oral glucose tolerance test and streptozotocin induced diabetic model in mice. Furthermore, *in-vitro* α -amylase and α-glucosidase enzyme assays also revealed the inhibition of these enzymes indicating the decrease in intestinal glucose absorption. Besides, twenty three phytocompounds were identified via HPLC and GCMS analysis. Among these compounds, myricetin, quercetin, rutin, and kaempferol showed the most probable anti-diabetic activity through enzyme inhibition (α-amylase, α-glucosidase), insulin secretion stimulation, enhanced insulin receptor kinase activity and PPARγ expression, and protection of the pancreas in QSAR analysis and network pharmacology study. Besides, *in silico*-based molecular docking and dynamic simulation studies strongly supported the *in vitro* and *in vivo* anti-diabetic effect of the experimental extract. Therefore, both the experimental and computational studies divulged the anti-diabetic effect of *C. quinquefolia.* However, further studies are needed to isolate the biologically active compound (s) as well as identify their molecular mechanism (s) of action in a diabetic model.

## 6 Ethics approval

All the *in vivo* experimental procedures were performed according to the ethical standards approved by the Ethical Review Committee, Faculty of Biological Science and Technology, Jashore University of Science and Technology [Ref: ERC/FBS/JUST/2022–111].

## Funding

This work was supported by the 10.13039/100020701Prime Minister's Education Assistance Trust Fellowship (Grant: 37.24.000.003.22.19). Also, the project was also supported by a grant from the 10.13039/501100016172Jashore University of Science and Technology (Grant: JUST/Research Cell-112/FoBST-05/2022-23). This study was also funded by Researchers Supporting Project number (RSPD2023R1035), 10.13039/501100021557King Saud University, Riyadh, Saudi Arabia.

## CRediT authorship contribution statement

**Nazmun Nahar:** Investigation, Data curation, Software, Validation, Writing – original draft, Visualization. **Md. Nazmul Hasan Zilani:** Conceptualization, Methodology, Formal analysis, Writing – review & editing, Visualization. **Partha Biswas:** Investigation, Software, Validation. **Md. Morsaline Billah:** Supervision. **Shabana Bibi:** Supervision, Funding acquisition. **Norah A. Albekairi:** Supervision, Funding acquisition. **Abdulrahman Alshammari:** Supervision, Funding acquisition. **Md. Nazmul Hasan:** Resources, Supervision, Project administration, Funding acquisition.

## Declaration of competing interest

The authors declare that they have no known competing financial interests or personal relationships that could have appeared to influence the work reported in this paper.

## Appendix A Supplementary material

Supplementary data 1 Figure S1: Binding of compounds with 3WY1. Left sides in Figure represent the 3D structure and the right side indicates a 2D structure of ligand-protein binding complexes. In 3D structure, ligand molecules were represented in golden colour with adjacent amino acid residues of 3WY1. Right side in Figure showed hydrogen bond in olive dotted green colour. Here, (A) was control drug Miglitol. Other compounds were (B) 12,3,4-tetrahydro-3-(phenylacetalatamido) quinoline, (C) Myricetin, (D) Quercetin. Figure S2: Binding of compounds with 6JB3. Left sides in Figure represent the 3D structure and the right side indicates a 2D structure of ligand-protein binding complexes. In 3D structure, ligand molecules were represented in golden colour with adjacent amino acid residues of 6JB3. Right side in Figure showed hydrogen bond in olive dotted green colour. Here, (A) was control drug Repaglinide. Other compounds were (B) Rutin, (C) Quercetin, (D) Kaempferol. Figure S3: Binding of compounds with 8B8Z. Left sides in Figure represent the 3D structure and the right side indicates a 2D structure of ligand-protein binding complexes. In 3D structure, ligand molecules were represented in golden colour with adjacent amino acid residues of 8B8Z. Right side in Figure showed hydrogen bond in olive dotted green colour. Here, (A) was control drug Repaglinide. Other compounds were (B) Rutin, (C)Z-23-dotriaconten-2-one, (D) Myricetin. Figure S4: The MolSA and PSA values for 1B2Y in complex with the five ligand compounds recovered from the complex system’s C atoms. Where blue, orange, grey, yellow and sky-blue colour represent the selected five ligand compounds. Figure S: The RMSD, RMSF, Rg, SASA, MolSA and PSA values for 3WY1 in complex with the five ligand compounds recovered from the complex system’s C atoms. Where blue, orange, grey, yellow and sky-blue colour represent the selected five ligand compounds. Figure S6: The RMSD, RMSF, Rg, SASA, MolSA and PSA values for 6JB3 in complex with the five ligand compounds recovered from the complex system’s C atoms. Where blue, orange, grey, yellow and sky-blue colour represent the selected five ligand compounds. Figure S7: The RMSD, RMSF, Rg, SASA, MolSA and PSA values for 8B8Z in complex with the five ligand compounds recovered from the complex system’s C atoms. Where blue, orange, grey, yellow and sky-blue colour represent the selected five ligand compounds. Figure S8: Representation of the 3WY1 protein-ligand interaction by 100 ns MD simulation. Figure S9: Representation of the 6JB3 protein-ligand interaction by 100 ns MD simulation. Figure S10: Representation of the 8B8Z protein-ligand interaction by 100 ns MD simulation. Figure S11: Network Pharmacology analysis of 3WY1 associated diabetes induced genes with the selected drug candidates. Here, (A) Construction of 3WY1network with the neighbouring genes based on mean interaction sources, (B) Construction of the drug-protein interactions (DPI) using STITCH and Cytoscape mapping to demonstrate multiple diabetes protein targets for the drug candidates. Figure S12: Network Pharmacology analysis of 6JB3 associated diabetes induced genes with the selected drug candidates. Here, (A) Construction of 6JB3 network with the neighbouring genes based on mean interaction sources, (B) Construction of the drug-protein interactions (DPI) using STITCH and Cytoscape mapping to demonstrate multiple diabetes protein targets for the drug candidates. Figure S13: Network Pharmacology analysis of 8B8Zassociated diabetes induced genes with the selected drug candidates. Here, (A) Construction of 8B8Z network with the neighbouring genes based on mean interaction sources, (B) Construction of the drug-protein interactions (DPI) using STITCH and Cytoscape mapping to demonstrate multiple diabetes protein targets for the drug candidates
