# Supplementary material for: Profiling of secondary metabolite and evaluation of anti-diabetic potency of Crotalaria quinquefolia (L): In-vitro, in-vivo, and in-silico approaches
Source: Saudi Pharm J. 2023 Nov 25;32(1):101887. doi: 10.1016/j.jsps.2023.101887 (PMC10711518; doi:10.1016/j.jsps.2023.101887)
Supplement: Supplementary data 1 — Figure S1: Binding of compounds with 3WY1. Left sides in Figure represent the 3D structure and the right side indicates a 2D structure of ligand-protein binding complexes. In 3D structure, ligand molecules were represented in golden colour with adjacent amino acid residues of 3WY1. Right side in Figure showed hydrogen bond in olive dotted green colour. Here, (A) was control drug Miglitol. Other compounds were (B) 12,3,4-tetrahydro-3-(phenylacetalatamido) quinoline, (C) Myricetin, (D) Quercetin. Figure S2: Binding of compounds with 6JB3. Left sides in Figure represent the 3D structure and the right side indicates a 2D structure of ligand-protein binding complexes. In 3D structure, ligand molecules were represented in golden colour with adjacent amino acid residues of 6JB3. Right side in Figure showed hydrogen bond in olive dotted green colour. Here, (A) was control drug Repaglinide. Other compounds were (B) Rutin, (C) Quercetin, (D) Kaempferol. Figure S3: Binding of compounds with 8B8Z. Left sides in Figure represent the 3D structure and the right side indicates a 2D structure of ligand-protein binding complexes. In 3D structure, ligand molecules were represented in golden colour with adjacent amino acid residues of 8B8Z. Right side in Figure showed hydrogen bond in olive dotted green colour. Here, (A) was control drug Repaglinide. Other compounds were (B) Rutin, (C)Z-23-dotriaconten-2-one, (D) Myricetin. Figure S4: The MolSA and PSA values for 1B2Y in complex with the five ligand compounds recovered from the complex system’s C atoms. Where blue, orange, grey, yellow and sky-blue colour represent the selected five ligand compounds. Figure S: The RMSD, RMSF, Rg, SASA, MolSA and PSA values for 3WY1 in complex with the five ligand compounds recovered from the complex system’s C atoms. Where blue, orange, grey, yellow and sky-blue colour represent the selected five ligand compounds. Figure S6: The RMSD, RMSF, Rg, SASA, MolSA and PSA values for 6JB3 in complex with the fi [file mmc1.docx]

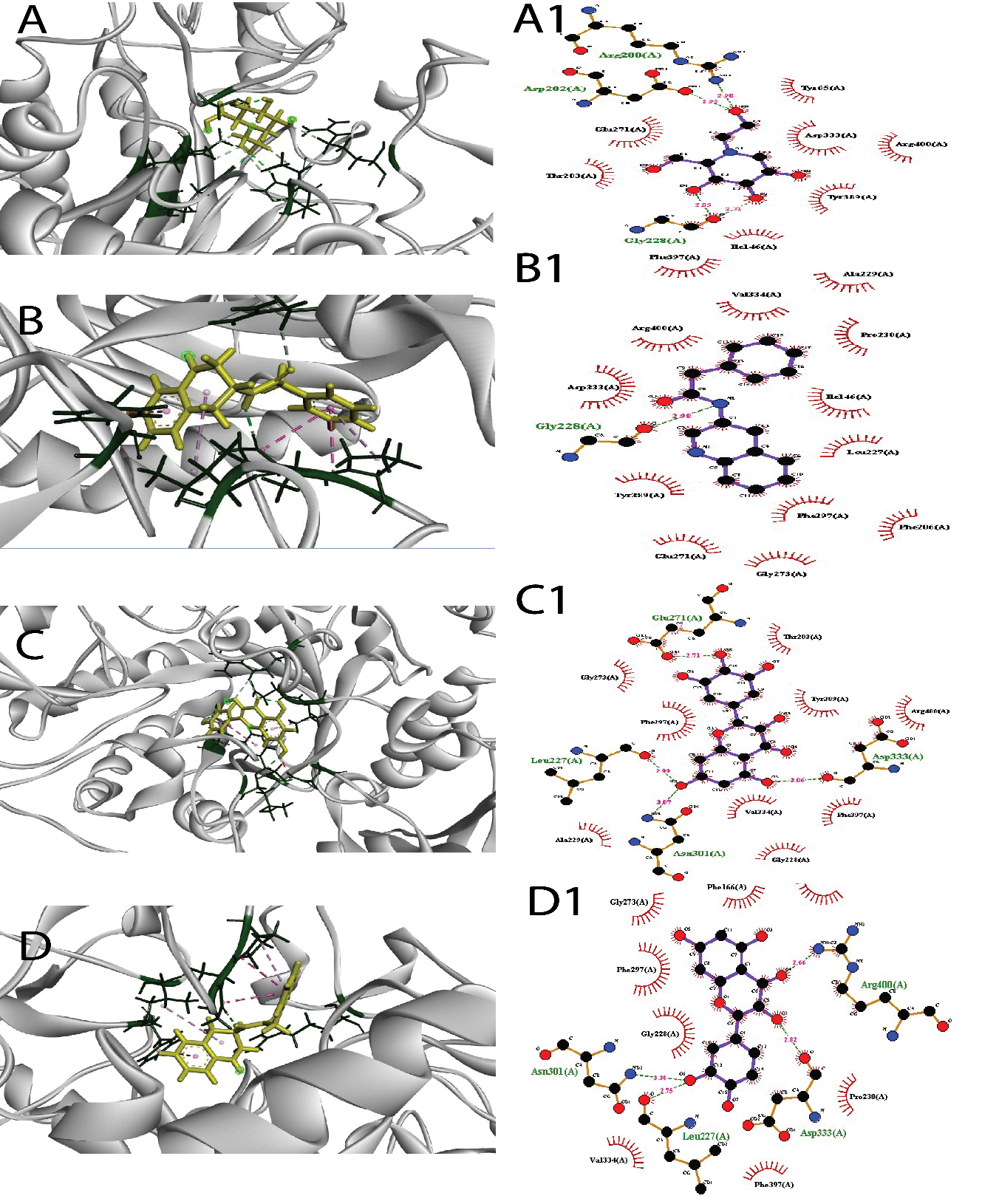


**Figure S1:** Binding of compounds with 3WY1. Left sides in figure represent the 3D structure and the right side indicates a 2D structure of ligand-protein binding complexes. In 3D structure, ligand molecules were represented in golden colour with adjacent amino acid residues of 3WY1. Right side in figure showed hydrogen bond in olive dotted green colour. Here, (**A**) was control drug Miglitol. Other compounds were (**B**) 12,3,4-tetrahydro-3-(phenylacetalatamido) quinoline, (**C)** Myricetin, (**D**) Quercetin.


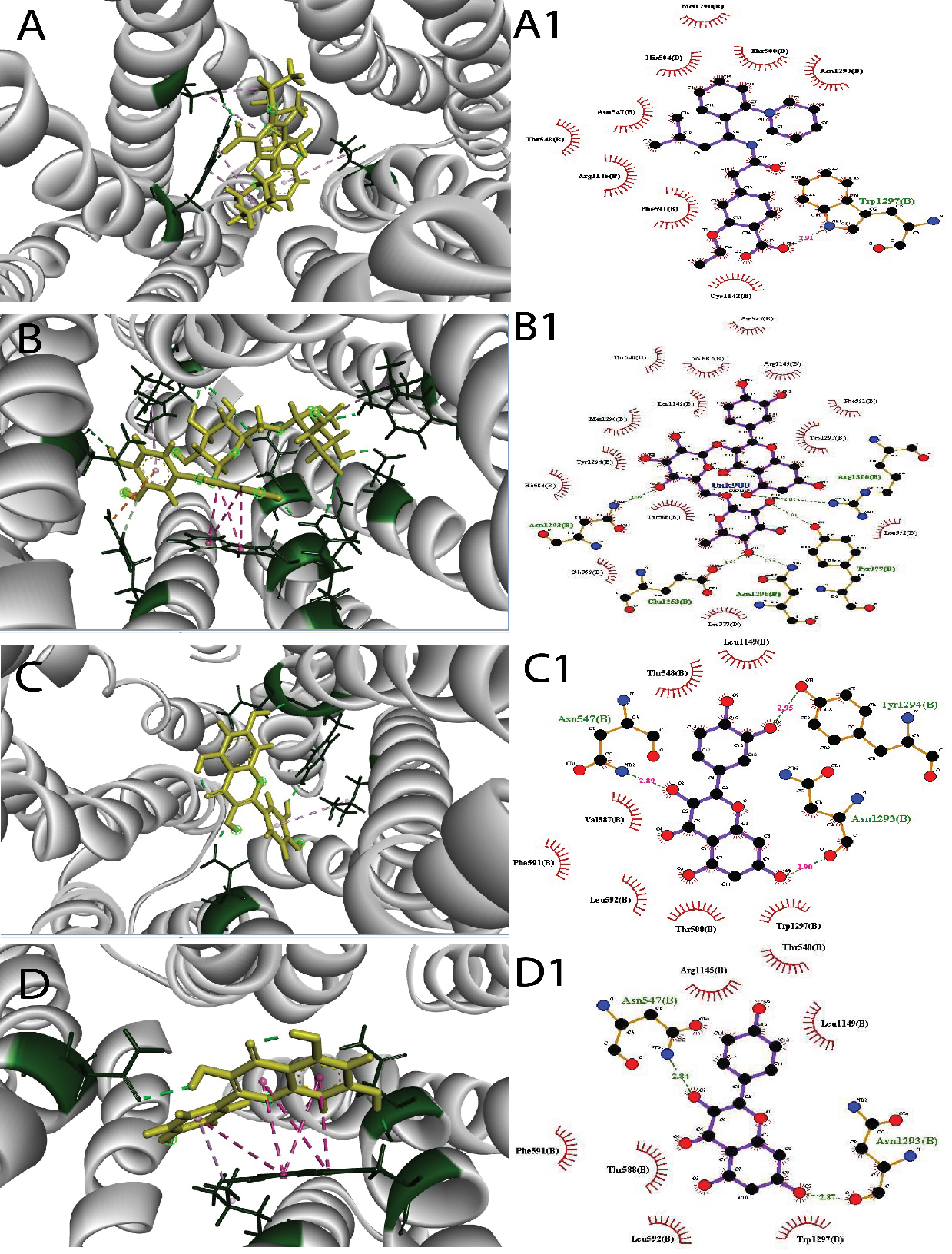


**Figure S2:** Binding of compounds with 6JB3. Left sides in figure represent the 3D structure and the right side indicates a 2D structure of ligand-protein binding complexes. In 3D structure, ligand molecules were represented in golden colour with adjacent amino acid residues of 6JB3. Right side in figure showed hydrogen bond in olive dotted green colour. Here, (**A**) was control drug Repaglinide. Others compounds were (B) Rutin, (**C)** Quercetin, (**D**) Kaempferol.


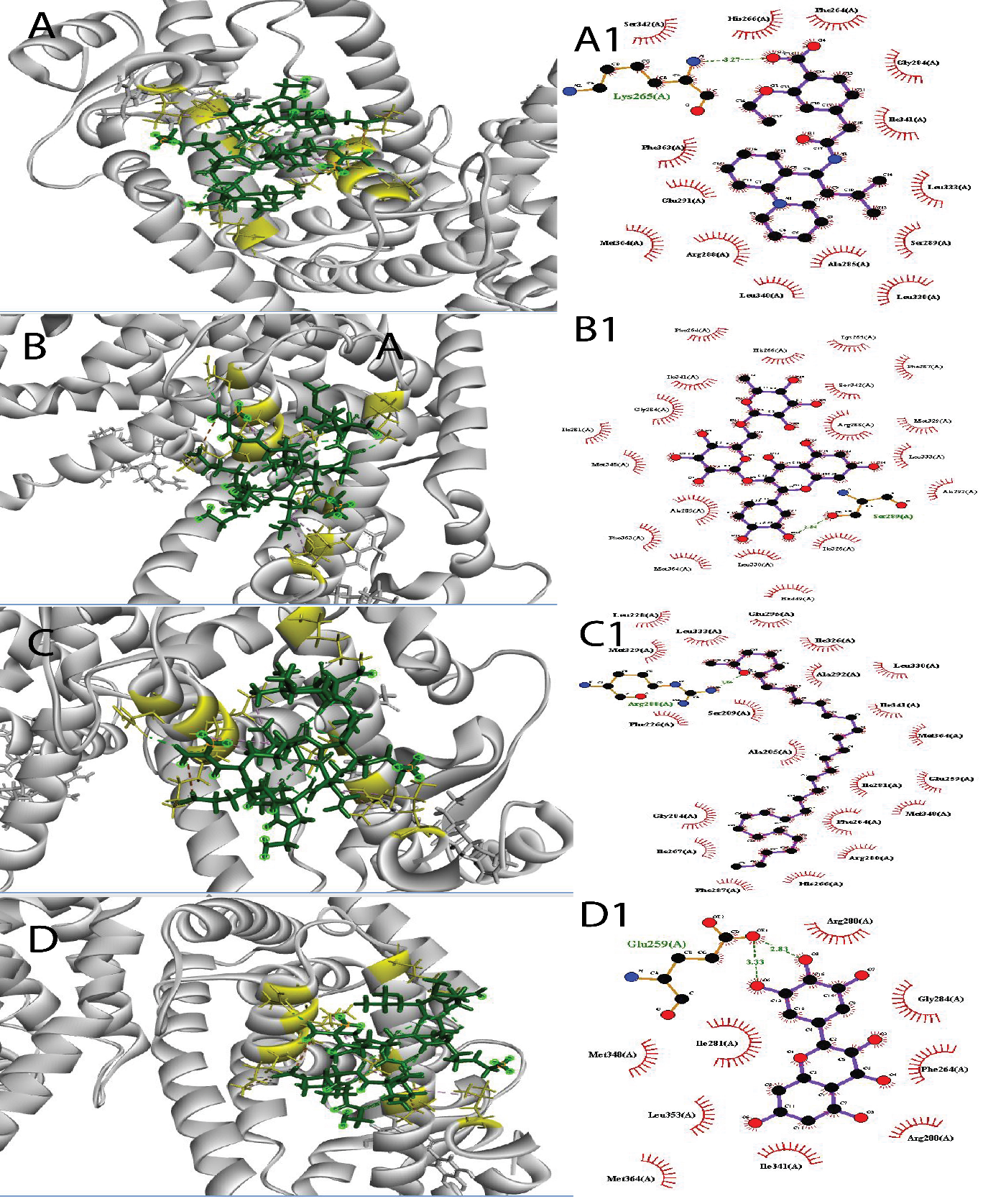


**Figure S3:** Binding of compounds with 8B8Z. Left sides in figure represent the 3D structure and the right side indicates a 2D structure of ligand-protein binding complexes. In 3D structure, ligand molecules were represented in golden colour with adjacent amino acid residues of 8B8Z. Right side in figure showed hydrogen bond in olive dotted green colour. Here, **(A)** was control drug Repaglinide. Others compounds were **(B)**Rutin, **(C)**Z-23-dotriaconten-2-one, (**D**) Myricetin.


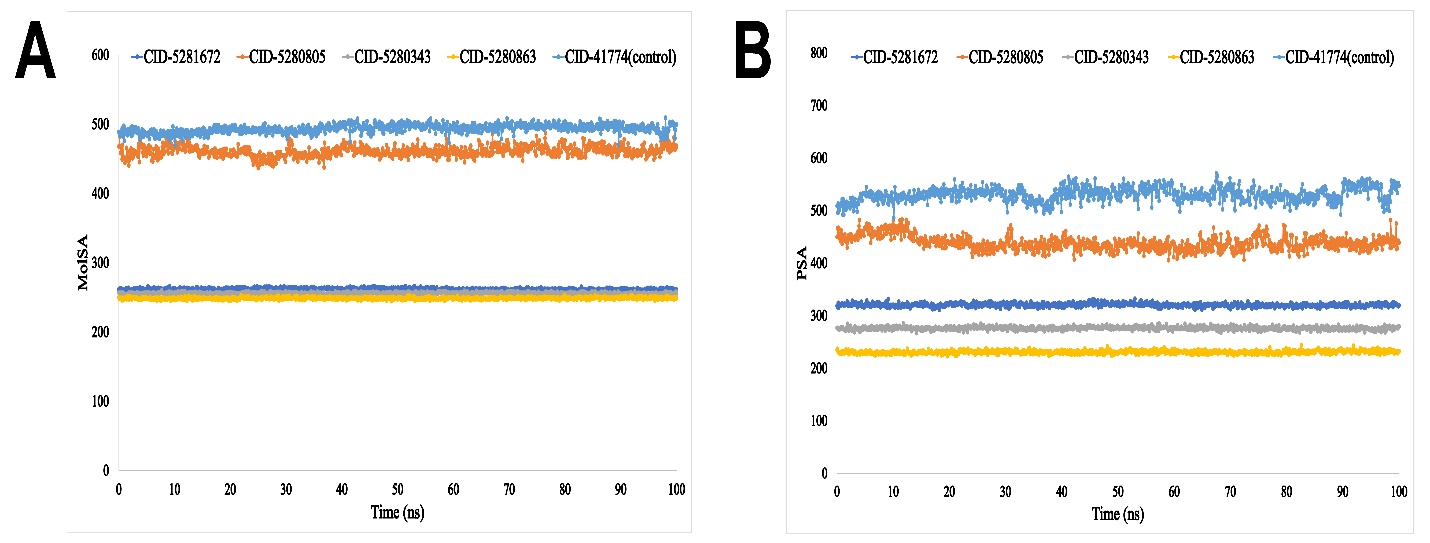


**Figure S4:** The MolSA and PSA values for 1B2Y in complex with the five ligand compounds recovered from the complex system’s C atoms. Where blue, orange, grey, yellow and sky-blue colour represent the selected five ligand compounds.


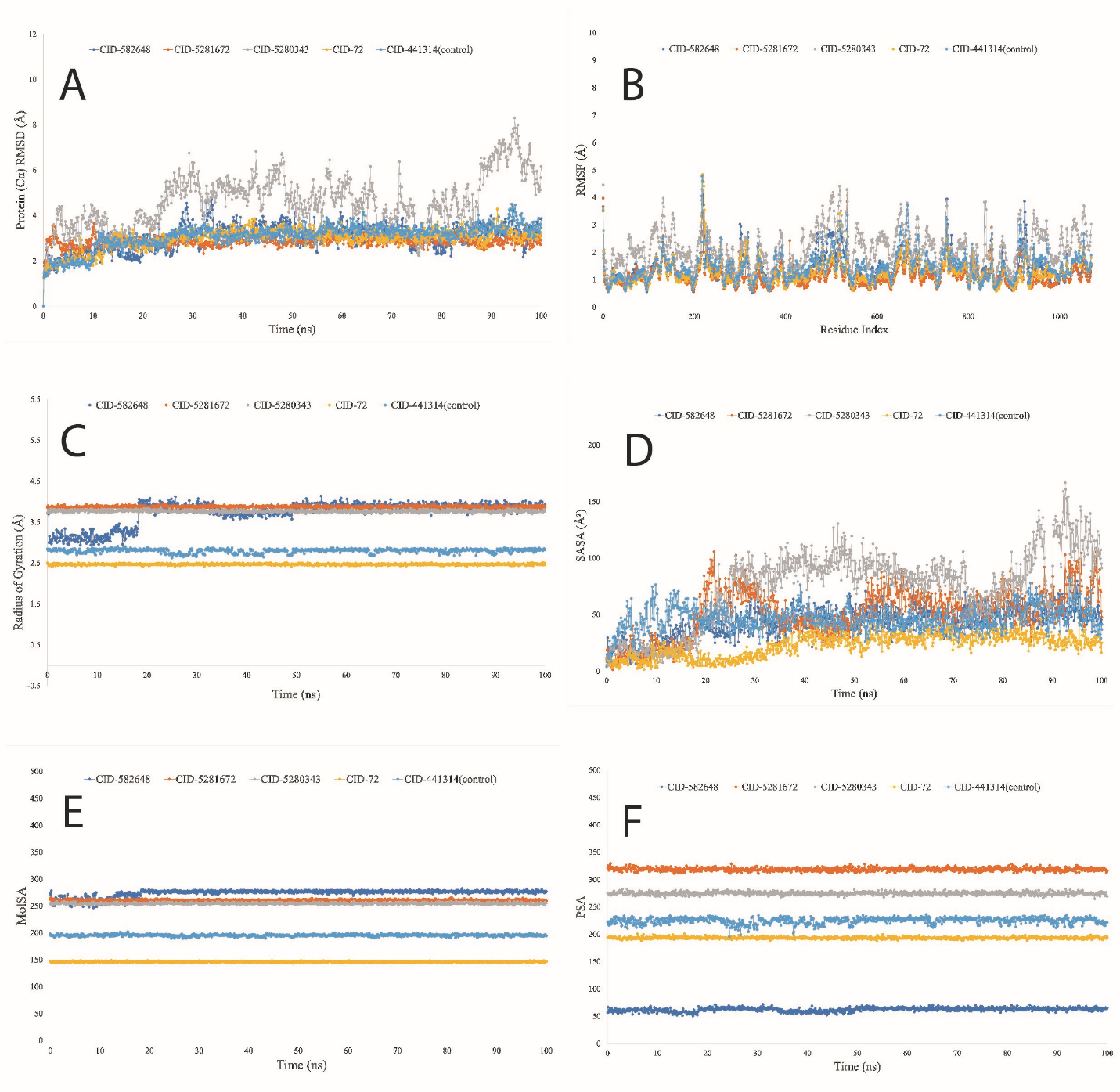


**Figure S5:** The RMSD, RMSF, Rg, SASA, MolSA and PSA values for 3WY1 in complex with the five ligand compounds recovered from the complex system’s C atoms. Where blue, orange, grey, yellow and sky-blue colour represent the selected five ligand compounds.


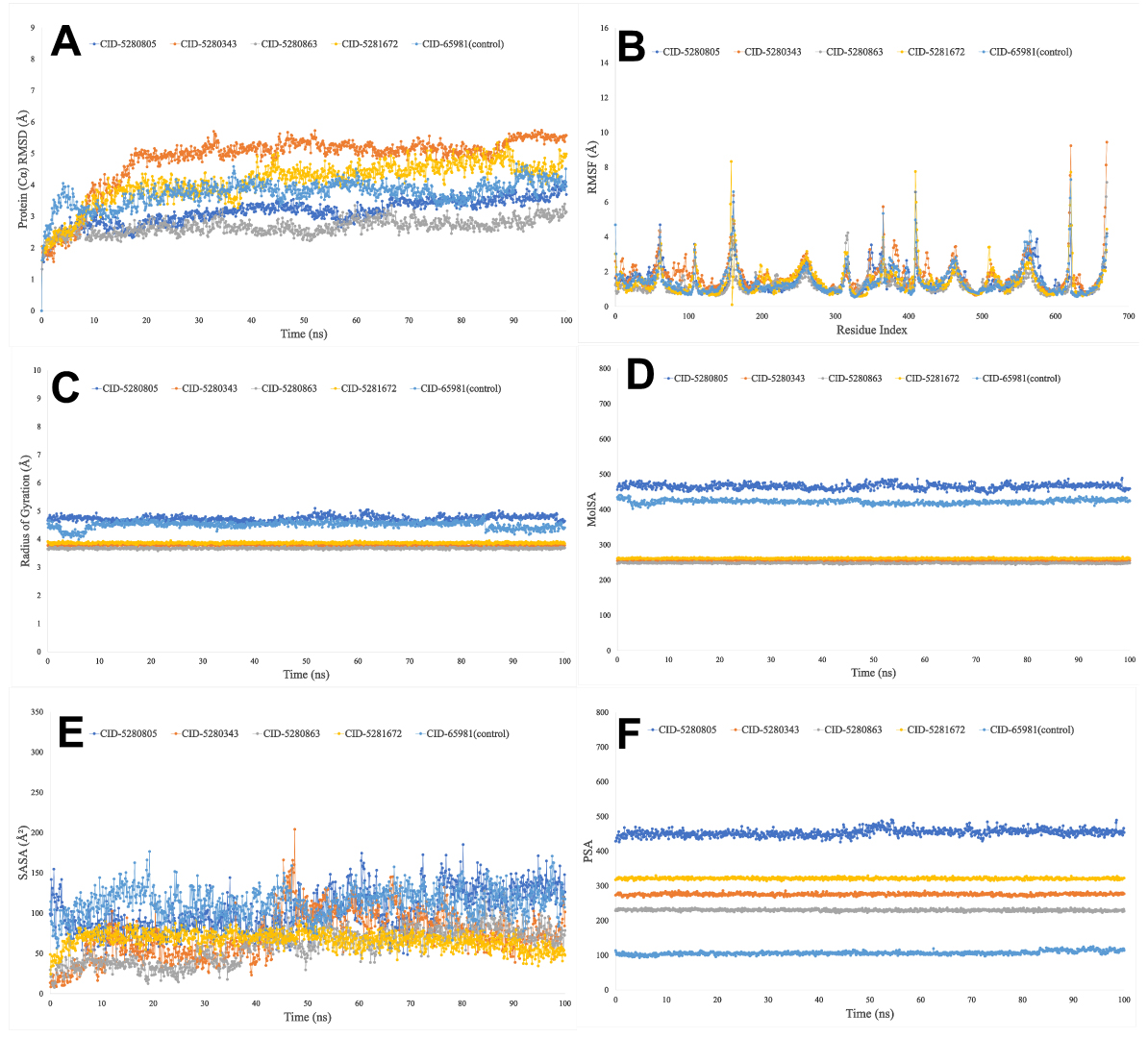


**Figure S6****:** The RMSD, RMSF, Rg, SASA, MolSA and PSA values for 6JB3 in complex with the five ligand compounds recovered from the complex system’s C atoms. Where blue, orange, grey, yellow and sky-blue colour represent the selected five ligand compounds.


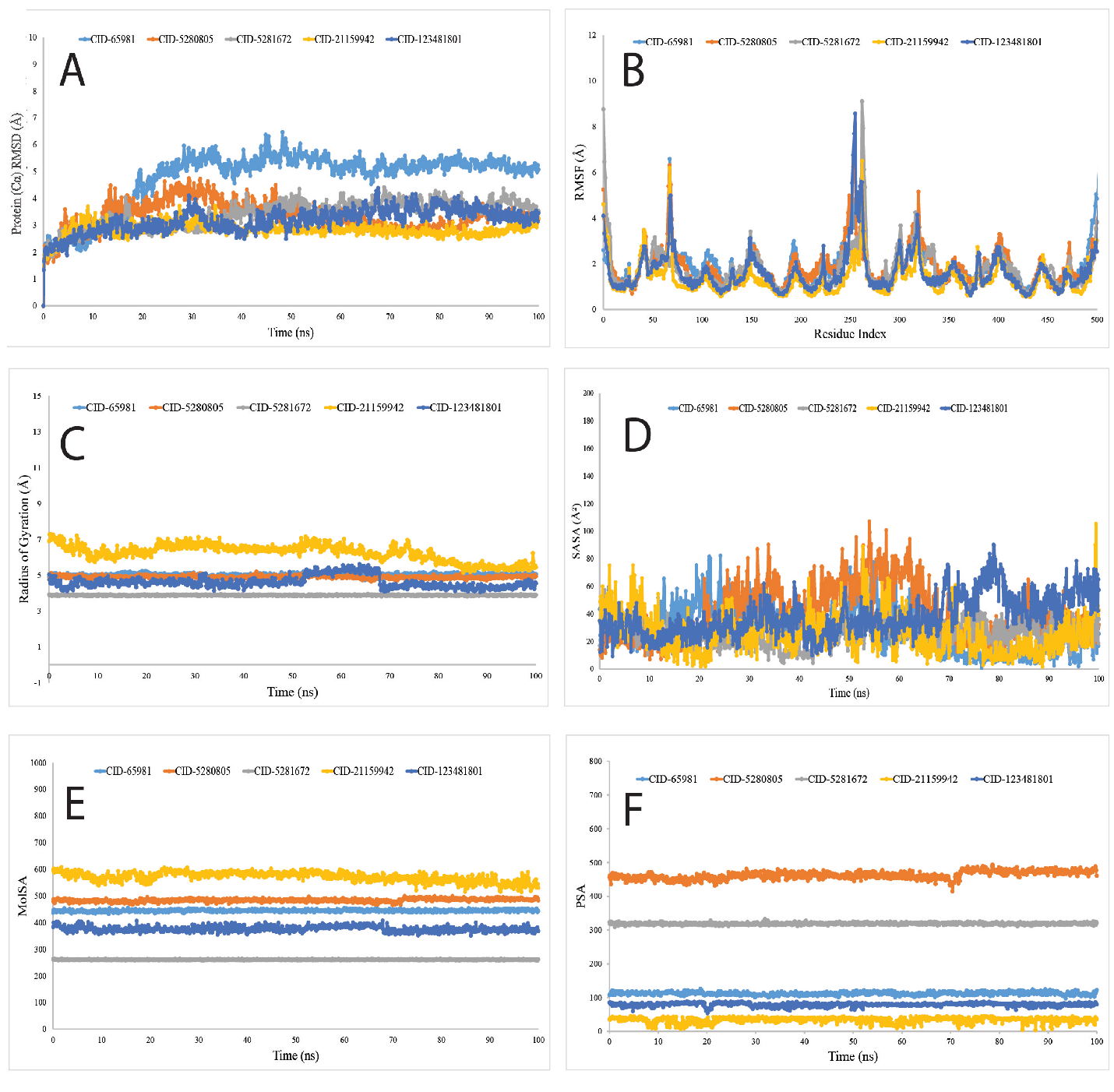


**Figure S7****:** The RMSD, RMSF, Rg, SASA, MolSA and PSA values for 8B8Z in complex with the five ligand compounds recovered from the complex system’s C atoms. Where blue, orange, grey, yellow and sky-blue colour represent the selected five ligand compounds.


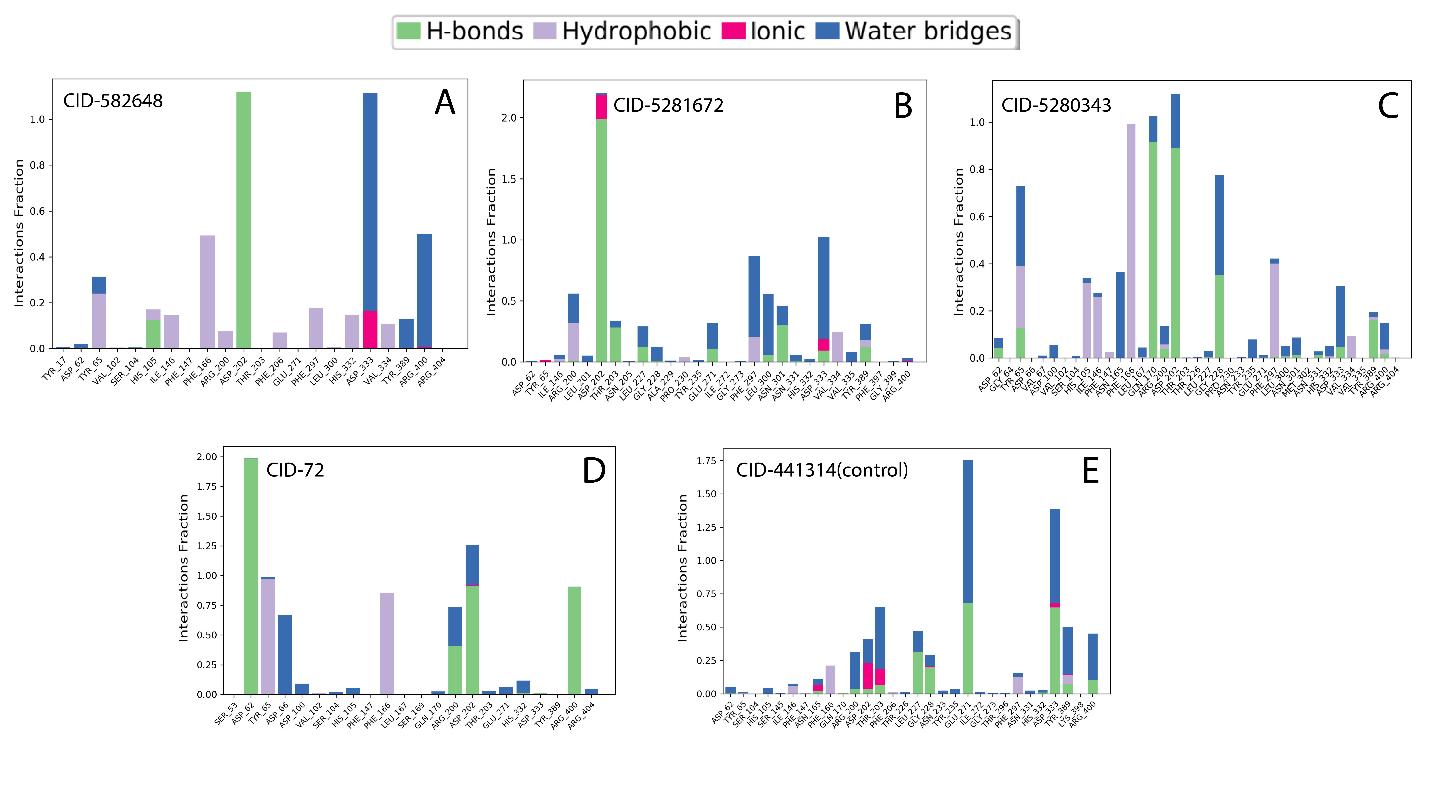
 **Figure S8:** Representation of the 3WY1 protein-ligand interaction by 100 ns MD simulation.


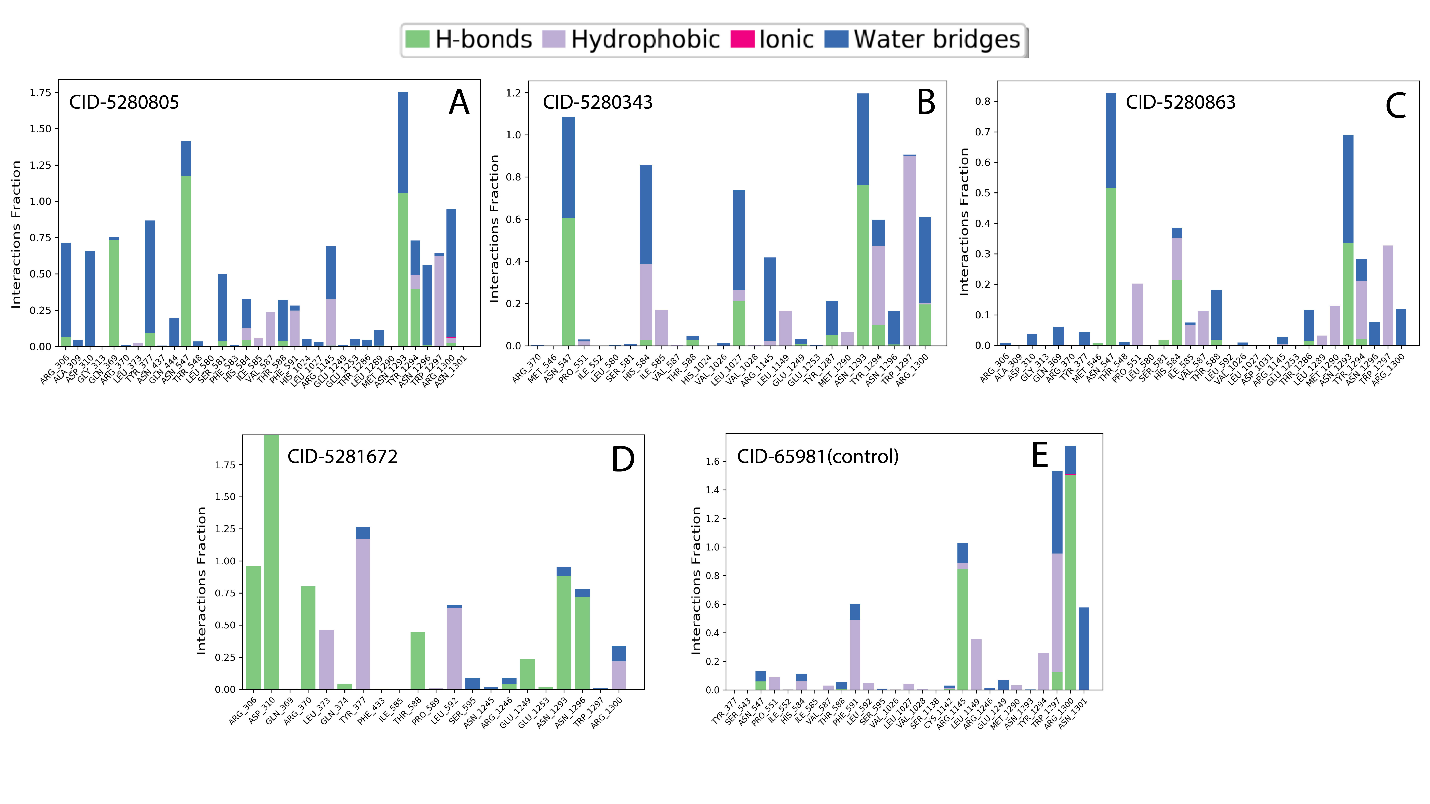
 **Figure S8:** Representation of the 6JB3 protein-ligand interaction by 100 ns MD simulation.


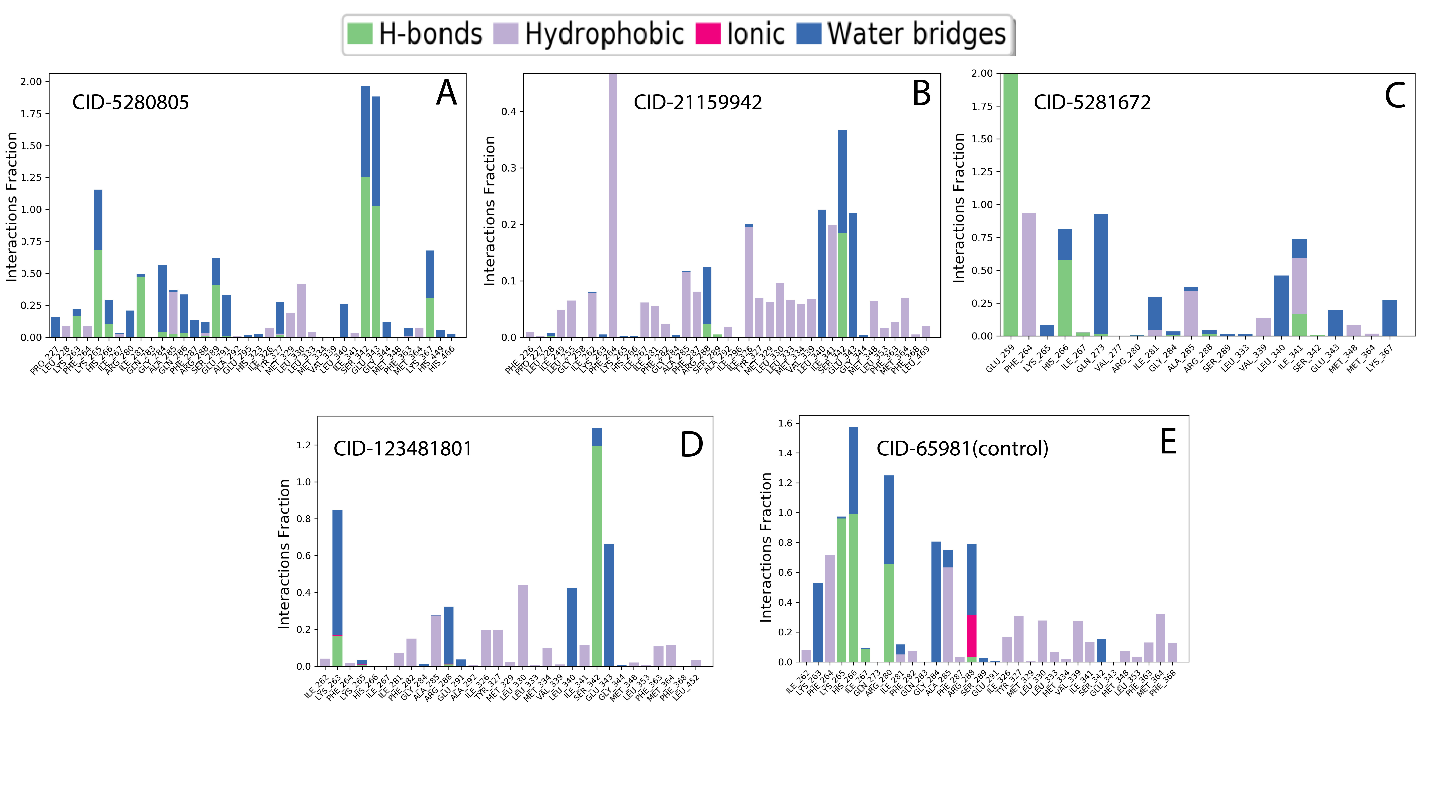
 **Figure S10:** Representation of the 8B8Z protein-ligand interaction by 100 ns MD simulation.


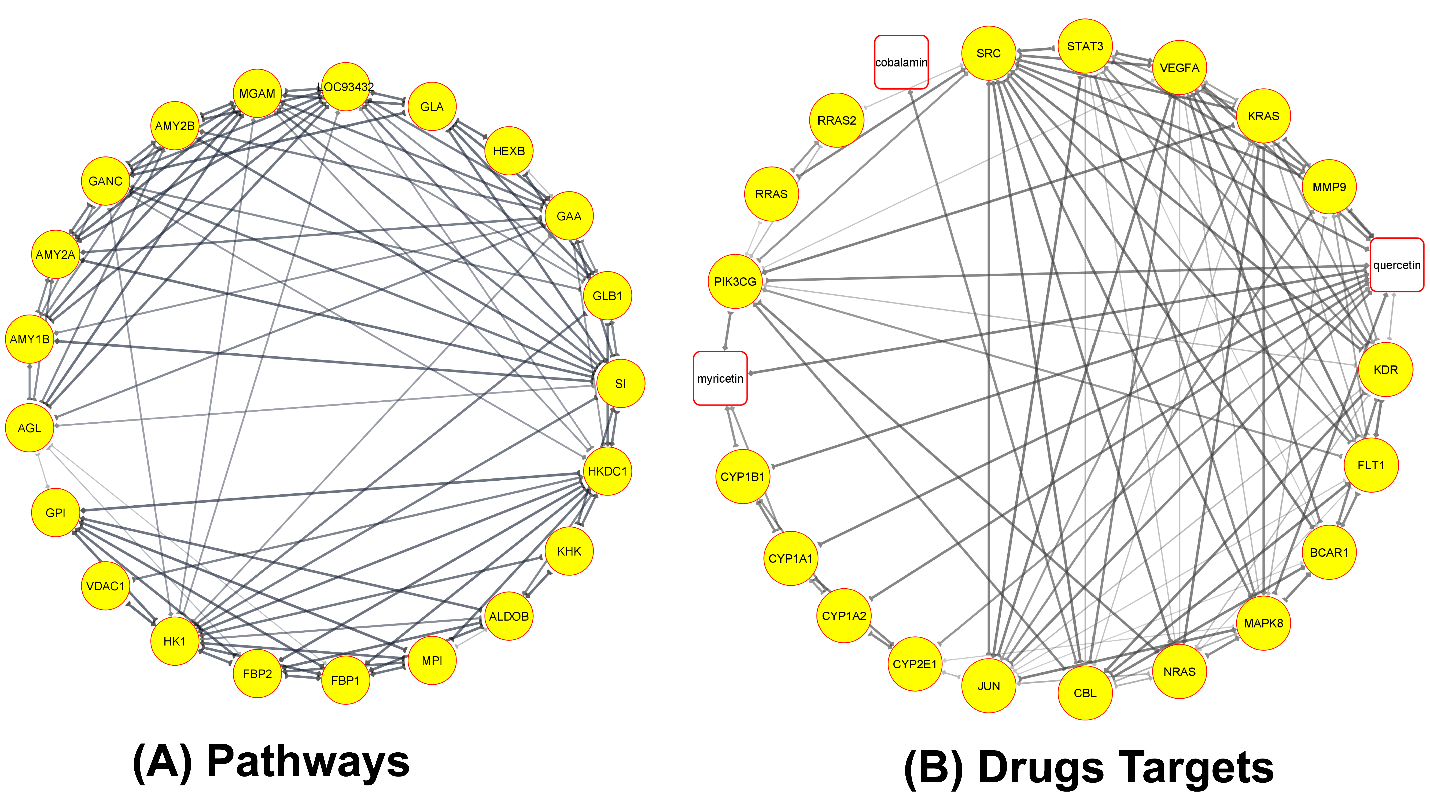


**Figure S11:** Network Pharmacology analysis of 3WY1 associated diabetes induced genes with the selected drug candidates. Here, (A) Construction of 3WY1network with the neighbouring genes based on mean interaction sources, (B) Construction of the drug-protein interactions (DPI) using STITCH and Cytoscape mapping to demonstrate multiple diabetes protein targets for the drug candidates.


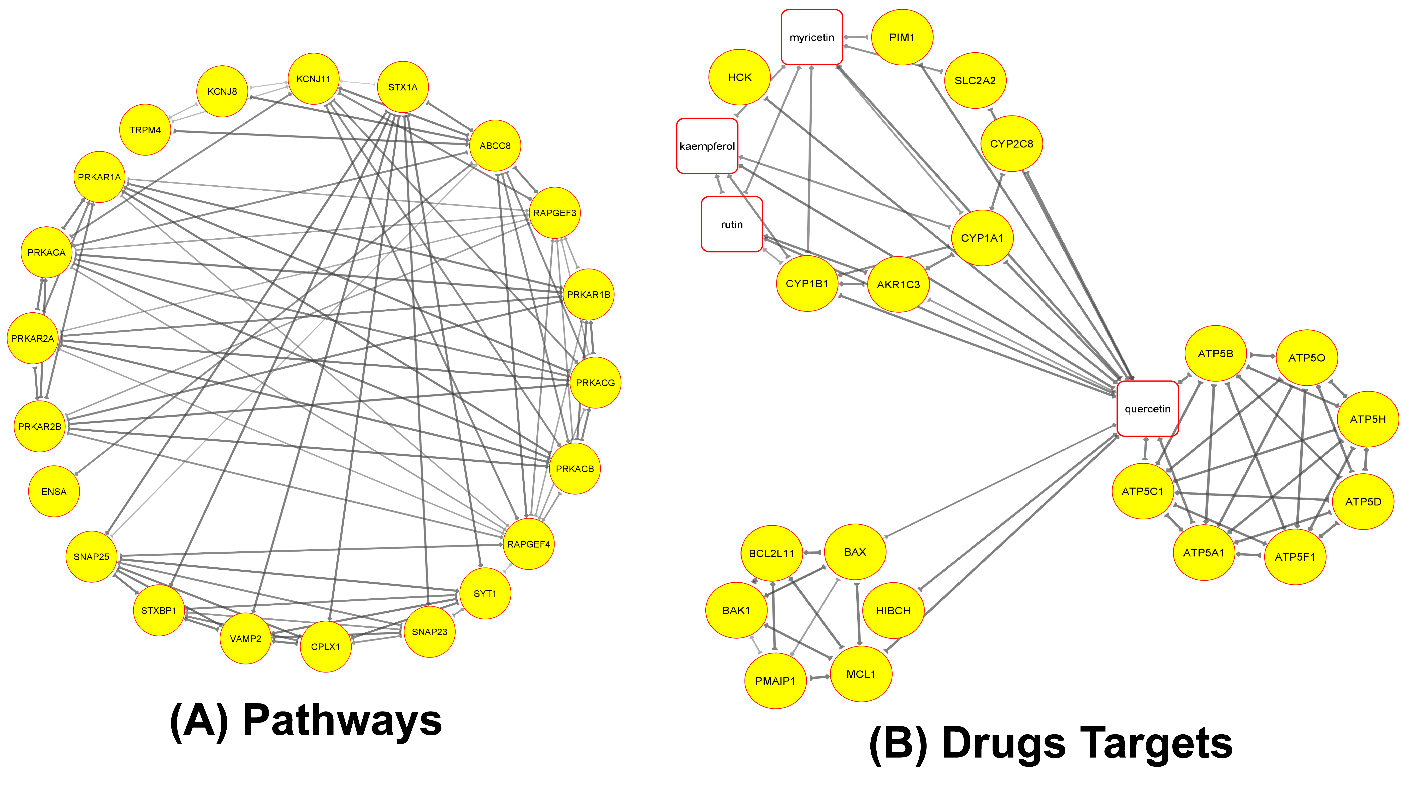
 **Figure S12:** Network Pharmacology analysis of 6JB3 associated diabetes induced genes with the selected drug candidates. Here, (A) Construction of 6JB3 network with the neighboring genes based on mean interaction sources, (B) Construction of the drug-protein interactions (DPI) using STITCH and Cytoscape mapping to demonstrate multiple diabetes protein targets for the drug candidates.

**Figure S13:** Network Pharmacology analysis of 8B8Zassociated diabetes induced genes with the selected drug candidates. Here, (A) Construction of 8B8Z network with the neighboring genes based on mean interaction sources, (B) Construction of the drug-protein interactions (DPI) using STITCH and Cytoscape mapping to demonstrate multiple diabetes protein targets for the drug candidates.
